# The pattern of anti-IL-6 versus non-anti-IL-6 biologic disease modifying anti-rheumatic drugs use in patients with rheumatoid arthritis in Wales, UK: a real-world study using electronic health records

**DOI:** 10.1093/rap/rkae140

**Published:** 2024-12-14

**Authors:** Roxanne Cooksey, Jonathan Kennedy, Muhammad Rahman, Sinead Brophy, Ernest Choy

**Affiliations:** CREATE Centre, Section of Rheumatology, Division of Infection and Immunity, School of Medicine, Cardiff University, Cardiff, UK; National Centre for Population Health and Wellbeing Research, Swansea, UK; CREATE Centre, Section of Rheumatology, Division of Infection and Immunity, School of Medicine, Cardiff University, Cardiff, UK; National Centre for Population Health and Wellbeing Research, Swansea, UK; Medical School, Swansea University, Health Data Research UK, Swansea, UK; Cardiff School of Technologies, Cardiff Metropolitan University, Cardiff, UK; National Centre for Population Health and Wellbeing Research, Swansea, UK; Medical School, Swansea University, Health Data Research UK, Swansea, UK; CREATE Centre, Section of Rheumatology, Division of Infection and Immunity, School of Medicine, Cardiff University, Cardiff, UK; National Centre for Population Health and Wellbeing Research, Swansea, UK

**Keywords:** rheumatoid arthritis, biologics, IL-6 inhibitors, infection

## Abstract

**Objective:**

Investigating factors associated with drug initiation and discontinuation in patients treated with anti-IL-6 biologic DMARDs (bDMARDs) (tocilizumab or sarilumab) *vs* non-anti-IL-6 (anti-TNF, B or T cell therapies) bDMARDs for RA.

**Methods:**

A retrospective cohort study of patients with the diagnosis of RA in the Secure Anonymised Information Linkage Databank, comprising primary and secondary care and specialist rheumatology clinic records for >90% of the population in Wales, UK. Patients initiated on first bDMARD treatment, discontinuation and clinical outcomes including infection and hospitalisation were analysed using Cox regression analysis.

**Results:**

Of patients identified with RA in their primary care records, 95.7% (4691/4922) received conventional synthetic DMARDs (csDMARDs). More than one-third (36.2%) were treated with bDMARDs (1784/4922). Of these biologic-naïve patients, 6.5% (116) were treated with anti-IL-6 bDMARDs; this treatment was associated with a previous history of infection [difference 8.8% (95% CI 1.1, 17.8)] and kidney disease [14.3% (95% CI 8.0, 22.5)]. Treatment discontinuation was significantly higher in the non-anti-IL-6 bDMARD-treated patients (23.1%) compared with the anti-IL-6 bDMARD-treated individuals (18.1%) [difference 9.4% (95% CI 1.1, 15.7)]. For those discontinuing a first line of treatment, 385 patients (23%) and 21 patients (18%) switched to an alternative bDMARD from the non-anti-IL-6 and anti-IL-6 groups, respectively.

**Conclusion:**

Comorbidities, history of infection and kidney disease were associated with choosing anti-IL-6 bDMARDs in biologic-naïve RA patients in Wales. Anti-IL-6 bDMARD-treated biologic-naïve patients were more likely to continue treatment than non-IL-6 bDMARD-treated patients.

Key messages

- Comorbidities, infection and kidney disease were associated with anti-IL-6 bDMARD use in biologic-naïve RA patients.
- Anti-IL-6 bDMARD-treated biologic-naïve patients were more likely to continue treatment than non-IL-6 bDMARD patients.
- Biologic treatment failure was associated with poorly controlled disease, highlighting the necessity for prompt treatment.

## Introduction

National and international RA management guidelines highlight the importance of early treatment to rapidly reduce disease activity and prevent long-term damage [1–4]. Today, biologic DMARDs (bDMARDs) and targeted synthetic DMARDs (tsDMARDs) have revolutionised treatment.

In the UK, RA is managed by rheumatology services and bDMARDs and csDMARDs are prescribed only by rheumatologists. The first line of treatments is csDMARDs. Monotherapy with csDMARDs such as methotrexate, leflunomide or sulfasalazine is advised within the first 3 months of persistent symptoms once the diagnosis is made. Additional tsDMARDs can be switched or added to the treatment regimen if csDMARD monotherapy is unsuccessful. The use of biologics is advocated when there is an inadequate response to csDMARDs and the disease is moderate or [5, 6] severe [as measured by a score >5.1 on the 28-joint DAS (DAS28)] [7, 8]. Patients who tolerate biologics and improve their DAS28 score by ≥1.2 points by 6 months fulfil the criteria to continue with treatment. Non-responders to biologic agents may switch to an alternative biologic with an evaluation of the risks and benefits on an individual basis.

With many biologic agents available to treat RA in the UK, rheumatologists must choose the most appropriate agent suitable for a patient or start with the least expensive drug where no clear indications are present [1]. For instance, adalimumab is recommended when a patient has extra-articular symptoms, such as uveitis [9–11] or ulcerative colitis [12, 13], while etanercept can be considered for individuals who are at increased risk of infections [14], including pre-existing hepatitis C infection [15, 16].

The risk of serious infection is an important consideration before starting biologic therapy, as infections account for significant morbidity and mortality in RA [17]. In fact, a UK study found that 8% of RA patients surveyed were hospitalised as a result of serious infection each year [18]. Factors associated with the increased risk of infection in RA patients include advancing age and the presence of comorbidities [19]. It has been reported that infection is the most common adverse event leading to biologic treatment discontinuation [20, 21].

Tocilizumab and sarilumab are anti-IL-6 receptor antibodies that are also effective in treating symptoms of RA and preventing the progression of structural damage [22–25] and are recommended when disease activity is severe and has not responded adequately to csDMARD therapy [1]. As with biologic medication, the risk of infections in RA patients treated with IL-6 inhibitors has also been reported [26]. For instance, a significantly increased infection risk for those treated with tocilizumab compared with etanercept [hazard ratio (HR) 1.2 (95% CI 1.01, 1.79)] has been observed. IL-6 inhibitor monotherapy also has efficacy superior to that of adalimumab [27].

Registry data from the British Society for Rheumatology Biologics Registry for RA (BSRBR-RA) [29], including 14 436 individuals prescribed biologics, compared remission and low disease activity (LDA) in patients treated with TNF inhibitors (TNFis). Positive predictors of sustained remission or LDA included not smoking, adalimumab (compared with other TNFis), greater patient global score, greater swollen joint count, more recent initiation of a TNFi and concomitant MTX. Poor baseline functional status, female gender, being older when commencing biologics, infliximab use, higher BMI and greater baseline ESR were negatively associated with sustained remission. Between 68% and 78% of patients did not achieve remission or LDA [30].

More information is required to assess the real-life predicted infection risk burden in RA patients using biologics [28]. Factors associated with commencing and discontinuing non-anti-IL-6 bDMARDs or anti-IL-6 bDMARDs are also of interest to guide rheumatologists in clinical practice when faced with choosing between many drugs available.

Here, using linked, routinely collected health data from the Secure Anonymised Information Linkage (SAIL) Databank, we prospectively and retrospectively follow patients through the healthcare system. By linking health data from two rheumatology services in Wales to hospital and primary care records, we assess the role of non-anti-IL-6 bDMARDs and anti-IL-6 bDMARDs (tocilizumab and sarilumab) in the management of RA in a real-world setting.

## Methods

### Data source

Routinely collected electronic health records from patients with RA were extracted and linked from the SAIL Databank [31]. The SAIL Databank holds more than one billion anonymised records. It uses a split-file approach to ensure anonymisation and overcome issues of confidentiality and disclosure in health-related data warehousing. Demographic data are sent to a partner organisation, the National Health Service Wales Informatics Service, where identifiable information is removed; clinical data are sent directly to the SAIL Databank and an individual is assigned an encrypted anonymised linkage field (ALF). The ALF is used to link anonymised individuals across datasets, facilitating longitudinal analysis of an individual’s journey through multiple health, education and social datasets. Data collected by physicians in primary care are captured using Read Codes (five-digit codes related to diagnosis, medication and process of care codes) [32]. Hospital inpatient and outpatient data are collected in the Patient Episode Database for Wales, which contains clinical information regarding patients’ hospital admissions, discharges, diagnoses and operations using the International Classification of Diseases, Tenth Revision (ICD-10) clinical coding system. The rheumatology clinic data contain information on rheumatological appointments, such as medications prescribed by rheumatologists and rheumatology assessments from two Health Board areas in Wales. Please see Supplementary Table S1, available at *Rheumatology Advances in Practice* online, for the variable data sources. Supplementary Tables S2 and S3, available at *Rheumatology Advances in Practice* online, include the Read Codes used for primary care for comorbidities and medications, respectively. Supplementary Table S4, available at *Rheumatology Advances in Practice* online, includes secondary care codes for comorbidities and Supplementary Table S5, available at *Rheumatology Advances in Practice* online, includes the operation codes for orthopaedic surgery.

Data were extracted from sources for the study period 2009–2020.

### Participants

RA patients ≥18 years of age at diagnosis were identified from the rheumatology dataset by ICD-10 codes present for the condition. For bDMARD-specific analysis, only those who received these medications were included.

### Exclusion criteria

Individuals were excluded from analysis if Read Codes for PsA or AS were present in the primary care data.

### Data linkage

Data were linked at the patient level to primary care and hospital admissions data to explore patient journey and health outcomes. Data were included from 2008 to 2020 to coincide with optimum data coverage/biologic prescription.

### Ethical approval

Data held in the SAIL Databank are anonymised and therefore no ethical approval was required. All data included had permission to be held in the SAIL Databank from the relevant Caldicott Guardians or Data Protection Officers. SAIL-related projects are required to obtain Information Governance Review Panel approval.

### Exposures of interest

The main exposures of interest were treatment with TNF blockers (etanercept, infliximab, golimumab, adalimumab and certolizumab pegol), abatacept (T cell modulator) or rituximab (anti-B cell) *vs* treatment with IL-6is (tocilizumab or sarilumab), which were obtained from the rheumatology dataset. Non-anti-IL-6 bDMARDs and anti-IL-6 bDMARDs were searched for by name in the rheumatology clinic data, as they were not always recorded by ICD-10 codes.

### Outcomes

Outcomes included initiation of non-anti-IL-6 bDMARDs or anti-IL-6 bDMARDs and time to treatment failure, as defined by stopping and switching to an alternative bDMARD.

### Covariates of interest and confounding factors

The baseline covariates considered were age, sex, BMI, level of social deprivation, disease duration, RF positivity and corticosteroid use, which were collected from the primary care records where relevant Read Codes were present or were calculated from available data (e.g. height and weight to calculate BMI). Please see Supplementary Table S1, available at *Rheumatology Advances in Practice* online, for all covariates, their data source and data type.

Comorbidities and medications were identified from Read Codes present in primary care health records, e.g. cardiovascular disease, diabetes, hyperlipidaemia, hypertension and steroids) (Supplementary Tables S2 and S3, available at *Rheumatology Advances in Practice* online). ICD-10 codes for orthopaedic surgery, infections and hospitalisations for infections from hospital admissions data were also included as covariates (Supplementary Tables S4 and S5, available at *Rheumatology Advances in Practice* online). Acute phase reactants, ESR and CRP measurements were unavailable.

### Statistical analysis

Descriptive statistics were used to examine covariate distribution at baseline. A Cox proportional hazards model was employed to calculate the HR of factors associated with treatment failure. Univariate analyses were performed to determine the significance of variables and a stepwise Cox proportional hazards model was used, which involved the selection of candidate variables to be included based on significance.

## Results

### Demographics

A total of 5058 individuals had codes for RA in their primary care data and were present in the South Wales secondary care rheumatology dataset. Individuals with Read Codes for PsA or AS were removed from the analysis (*n* = 136), resulting in a cohort of 4922. The mean age of the cohort was 62 years (s.d. 13.9), with a mean disease duration of 6.3 years (s.d. 2.9). Of these, 71.6% were female (3522/4922). The mean BMI of the sample was 28.1 (s.d. 6.0) (Table 1).

**Table 1.** Patient profiles of biologic-naïve RA patients taking non-anti-IL-6 bDMARDs or anti-IL-6 bDMARDs

Characteristics	Non-anti-IL-6 bDMARDs (*n* = 1668)	Anti-IL-6 bDMARD initiated (*n* = 116)	Difference (95% CI)
Age at diagnosis, years, mean (s.d.)	58.6 (13.2)	56.9 (13.2)	1.7 (−0.8, 4.2)
Disease duration, years, mean (s.d.)	7.2 (2.6)	6.1 (2.1)	1.1 (0.6, 1.6)
Female, % (*n*)	74.9 (1250)	75.0 (87)	0.1 (−7.4, 8.8)
BMI, mean (s.d.)	27.5 (5.6)	30.5 (6.4)	3.0 (0.6, 5.4)
Living in a rural area, % (*n*)	15.5 (257)	14.7 (17)	0.8 (−7.0, 6.4)
Ever smoked, % (*n*)	10.9 (181)	12.9 (15)	2.0 (−3.1, 9.5)
Time to treatment from diagnosis, years, mean (s.d.)	1.4 (2.3)	2.2 (2.4)	0.8 (0.4, 1.2)
Age at start of treatment, years, mean (s.d.)	60 (13.0)	59.0 (12.8)	1.0 (−1.4, 3.4)
Time to treatment from DMARD, years, mean (s.d.)	9.1 (10.6)	8.4 (6.5)	0.7 (−1.3, 2.7)
Number of csDMARDs pre-treatment, mean (s.d.)	1.9 (0.9)	1.9 (0.9)	0.0 (−0.2, 0.2)
bDMARD treatment change/fail, % (*n*)	23.1 (385)	18.1 (21)	5.0 (−3.3, 11.3)
Infections pre-treatment, % (*n*)	19.7 (328)	28.5 (33)	8.8 (1.1, 17.8)*
Infections post-treatment, % (*n*)	88.1 (1469)	77.6 (90)	10.5 (3.7, 19.0)*
Orthopaedic surgery pre-treatment, % (*n*)	33.9 (566)	29.3 (34)	4.6 (−4.5, 12.5)
Orthopaedic surgery post-treatment, % (*n*)	15.1 (252)	5.2 (6)	9.9 (4.1, 13.3)*
Kidney disease pre-treatment, % (*n*)	5.5 (92)	19.8 (23)	14.3 (8.0, 22.5)*
Diabetes pre-treatment, % (*n*)	8.4 (140)	14.7 (17)	6.3 (0.8, 13.9)
Hyperlipidaemia pre-treatment, % (*n*)	8.1 (135)	11.2 (13)	3.1 (−1.6, 10.2)
Hypertension pre-treatment, % (*n*)	31.6 (527)	30.2 (35)	1.4 (−7.7, 9.4)
Cardiovascular disease pre-treatment, % (*n*)	12.3 (205)	9.5 (11)	2.8 (−4.1, 7.2)
Steroid use, % (*n*)	77.3 (1289)	75 (87)	2.3 (−5.0, 11.1)

* *P* < 0.05.

### Medication use

**Figure 1.**
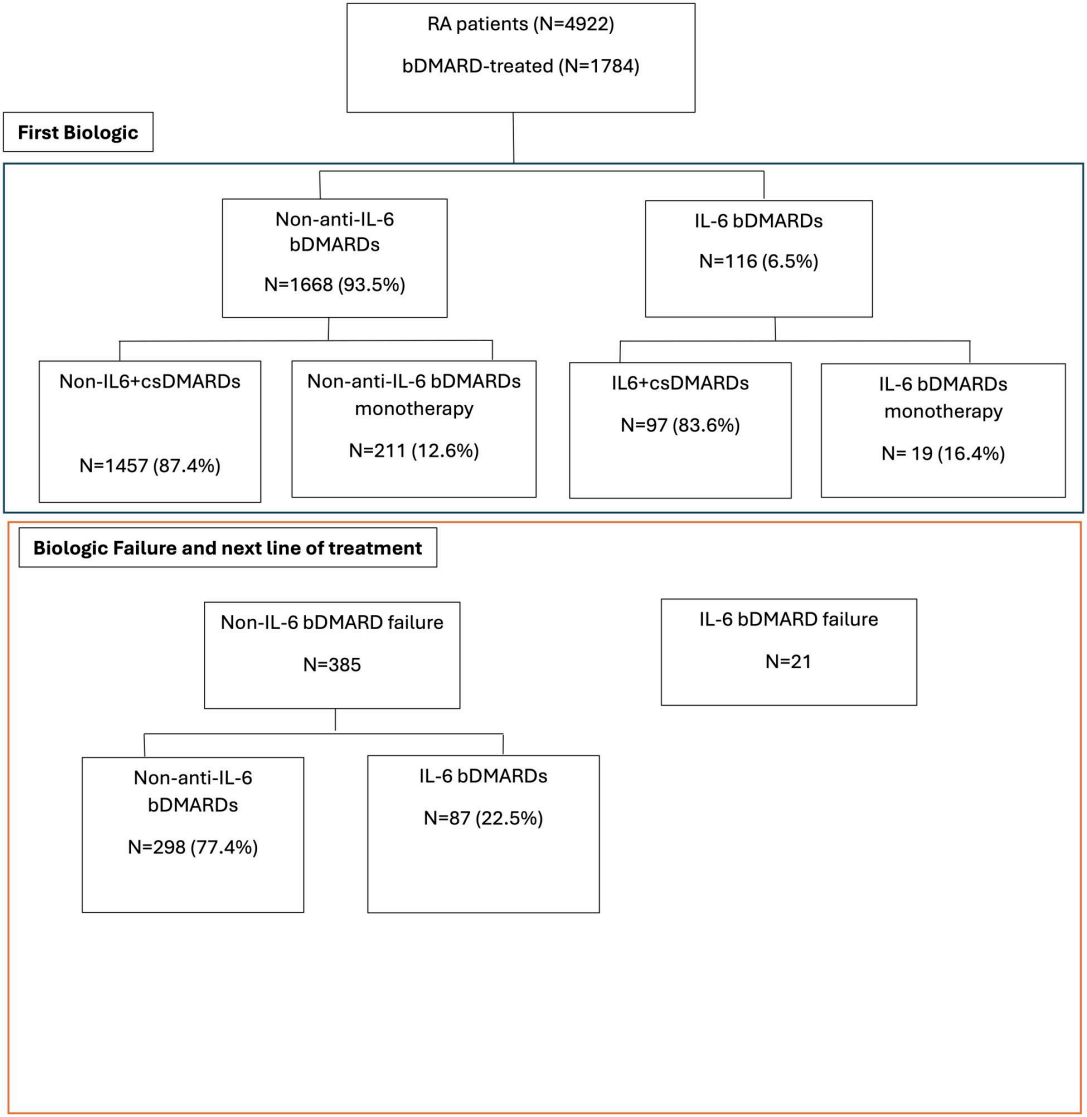
Medication status of RA patients in the SAIL Databank

Of all the patients, 95.7% (4691/4922) had taken a csDMARD. Of these, 29.6% also took bDMARDs for their RA (1457/4922) and they took on average 1.9 (s.d. 0.9) csDMARDs before commencing biologic therapy (Fig. 1 and Table 1).

Individuals who received csDMARDs before taking the anti-IL-6 bDMARDs tocilizumab or sarilumab [2% (97/4922)] took on average 1.9 (s.d. 0.9) csDMARDs before starting the treatment (Fig. 1 and Table 1). The overall rate of anti-IL-6 bDMARD use in the cohort was 5.7%, although just 2% (97/4922) took this as the first line of therapy following csDMARDs. For non-IL-6-bDMARD use, 15.2% (747/4922) used etanercept, 11.6% rituximab (570/4922) and 7.6% (376/4922) adalimumab.

**Figure 2.**
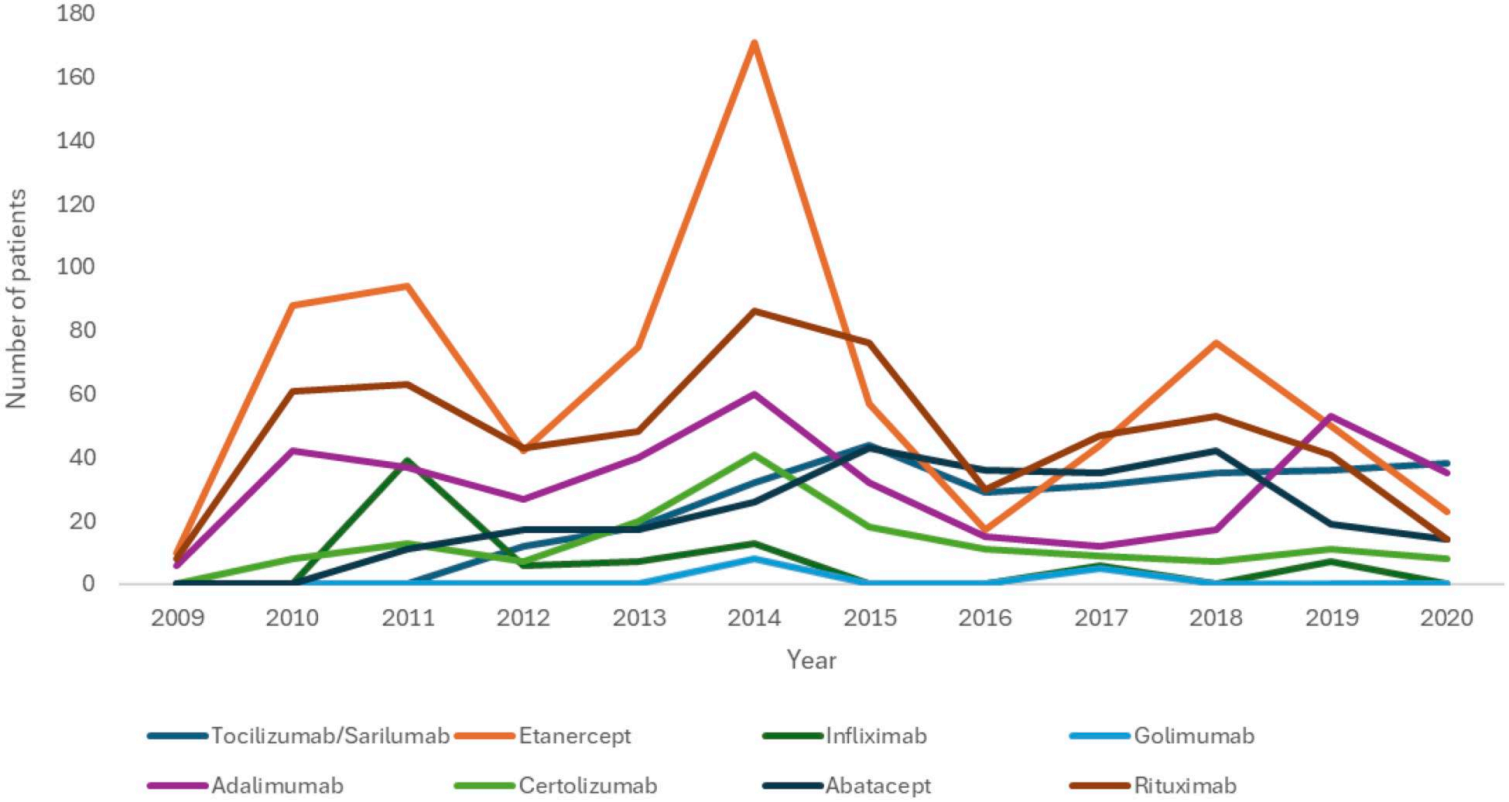
Period prevalence of RA patients treated with biologics and IL-6 inhibitors in Wales for the period 2009–2020

Figure 2 presents the use of non-anti-IL-6 and anti-IL-6 bDMARD therapy from 2009 to 2020. Non-anti-IL-6 bDMARD use was at its highest in 2014, particularly treatment with etanercept, rituximab and adalimumab. The use of non-anti-IL-6 bDMARDs included in this analysis decreased from 2014 to 2016 and began to increase again in 2018 and 2019, followed by a decline in 2020.

For anti-IL-6 bDMARDs, use began in 2011, peaked in 2015 and decreased in 2016. This was followed by a period of increased use from 2017 to 2020 (Fig. 2). A total of 27.5% (458/1668) [difference 9.4% (95% CI 1.1, 15.7)] of patients added (first initiation of biologic), switched (change from one biologic to another) or stopped a prescribed biologic treatment (Table 1).

### Factors associated with treatment with a bDMARD

In the final model, the Cox proportional hazards model showed that treatment with biologic therapy was associated with an incremental increase in disease duration per year [HR 1.11 (95% CI 1.07, 1.15)]. Younger age was associated with a decremental decrease in initiation of biologic therapy per year [HR 0.98 (95% CI 0.97, 0.98)]. Orthopaedic surgery was associated with a reduced use of biologics [HR 0.27 (95% CI 0.15, 0.49)], as was a kidney disease [HR 0.35 (95% CI 0.25, 0.48)] (Table 2).

**Table 2.** Final model of HRs of factors associated with being treated with non-anti-IL-6 bDMARDs in biologic-naïve RA patients

Variables	HR	95% CI	*P*-value
Increasing age at diagnosis (per year)	0.98	0.97, 0.98	<0.001*
Disease duration (per year)	1.11	1.07, 1.15	<0.001*
Orthopaedic surgery (pre-treatment)	0.27	0.15, 0.49	<0.001*
Kidney disease (pre-treatment)	0.35	0.25, 0.48	<0.001*

* *P* < 0.05 (see Supplementary Table S3, available at *Rheumatology Advances in Practice* online for univariate analysis).

### Factors associated with treatment with anti-IL-6 bDMARDs

Compared with non-anti-IL-6 bDMARDs, the rate of infections recorded as ICD-10 codes in secondary care data was significantly higher in anti-IL-6 bDMARD-treated patients before treatment [difference 8.8% (95% CI 1.1. 17.8)] (Table 1). The difference was statistically significant in the Cox proportional hazards model [HR 1.73 (95% CI 1.15, 2.59)] (Table 3). Yet following treatment, the rates of infection were significantly lower in these patients compared with non-IL-6 bDMARD-treated patients [difference 10.5% (95% CI 3.7, 19)] (Table 1).

**Table 3.** Final model of HR of factors associated with being treated with IL-6 inhibitors in biologic-naïve RA patients

Variable	HR	95% CI	*P*-value
Infections (pre-treatment)	1.73	1.15, 2.59	0.008*

* *P* < 0.05 (see Supplementary Table S4, available at *Rheumatology Advances in Practice* online for univariate analysis).

### Treatment failure

The rate of treatment failure (or change of treatment regimen) was 23.1% (385/1668) and 18.1% (21/116) for non-anti-IL-6 bDMARD- and anti-IL-6 bDMARD-treated individuals, respectively (Table 1). Non-anti-IL-6 bDMARD treatment failure was associated with orthopaedic surgery pre-biologic treatment [HR 1.64 (95% CI 1.00, 2.68)] and steroid use [HR 1.62 (95% CI 1.26, 2.08)]. Younger age at the time of diagnosis was associated with a lower risk of non-anti-IL-6 bDMARD failure [HR 0.99 (95% CI 0.98, 0.99)] (Table 4). Please see Supplementary Tables S6–S8, available at *Rheumatology Advances in Practice* online, for all univariate analysis that informed the candidate variables for Cox proportional hazards model building. There was no factor associated with the treatment failure of anti-IL-6 bDMARD-treated patients that was statistically significant in the model (Supplementary Table S9, available at *Rheumatology Advances in Practice* online).

**Table 4.** Final model of HR of time to treatment failure from first biologic

Variables	HR	95% CI	*P*-value
Older age at diagnosis (per year)	0.99	0.98, 0.99	<0.001
Orthopaedic surgery (pre-treatment)	1.64	1.00, 2.68	0.048*
Steroid use	1.62	1.26, 2.08	<0.001

* *P* < 0.05 (See Supplementary Table S5, available at *Rheumatology Advances in Practice* online for univariate analysis).

Of those discontinuing the first line of treatment, 385 patients (23%) and 21 patients (18%) switched to an alternative bDMARDs from the non-anti-IL-6 and anti-IL-6 groups, respectively (Table 1). Of the 385 switchers from the non-anti-IL-6 bDMARDs group, 298 patients (77.4%) received a second non-anti-IL-6 bDMARD and 87 patients received an anti-IL-6 bDMARD (22.5%). Treatment failure in biologic-experienced patients was significantly higher in the anti-IL-6 bDMARD-treated group [difference 28.3% (95% CI 17.6, 39.3)] (Table 5). The patient characteristics were not significantly different between the two groups (Supplementary Table S10, available at *Rheumatology Advances in Practice* online). However, concomitant hypertension, hyperlipidaemia and steroids use were numerically higher in the anti-IL-6 bDMARDs group, although the differences were not statistically significant.

**Table 5.** Patient profiles of bDMARD-experienced RA patients taking second-line treatment of either non-IL-6 or anti-IL-6 bDMARDs

Characteristics	Non-anti-IL-6 bDMARDs initiated (*n* = 298)	Anti-IL-6 bDMARDs initiated (*n* = 87)	Difference (95% CI)
Age diagnosis, years, mean (s.d.)	57.3 (12.0)	55.4 (12.1)	1.9 (−1.0, 4.8)
Disease duration, years, mean (s.d.)	8.1 (2.3)	8.4 (2.2)	0.3 (−0.9, 0.3)
Female, % (*n*)	76.5 (228)	80.5 (70)	4.0 (−12.6, 6.6)
BMI, % (*n*)	27.3 (6.2)	29.0 (6.6)	1.7 (−4.5, 1.1)
Ever smoked, % (*n*)	13.1 (39)	6.9 (6)	6.2 (−1.9, 11.9)
Biologic treatment change/fail, % (*n*)	13.1 (39)	41.4 (36)	28.3 (17.6, 39.3)*
Infections pre-treatment, % (*n*)	14.4 (43)	12.6 (11)	1.8 (−7.5, 8.8)
Infections post-treatment, % (*n*)	92.3 (275)	90.8 (79)	1.5 (−4.3, 9.8)
Orthopaedic surgery pre-treatment, % (*n*)	33.9 (101)	33.3 (29)	0.6 (−11.1, 11.1)
Orthopaedic surgery post-treatment, % (*n*)	17.8 (53)	25.3 (22)	7.5 (−1.8, 18.3)
Diabetes pre-treatment, % (*n*)	9.1 (27)	8.1 (7)	10 (−7.1, 6.6)
Hyperlipidaemia pre-treatment, % (*n*)	7.1 (21)	9.2 (8)	2.1 (−10.4, 3.5)
Hypertension pre-treatment, % (*n*)	28.5 (85)	32.2 (28)	3.7 (−15.1, 6.7)
Cardiovascular disease pre-treatment, % (*n*)	13.1 (39)	<5	–
Steroid use, % (*n*)	81.9 (244)	89.7 (78)	7.8 (−12.9, 14.6)

* *P* < 0.05.

### Sensitivity analyses

From sensitivity analysis investigating treatment failure in biologic-experienced patients, no significant factors were identified (Supplementary Table S11, available at *Rheumatology Advances in Practice* online). This analysis was not repeated for the tocilizumab-/sarilumab-treated patients due to low numbers.

## Discussion

This study found that the vast majority of patients under the care of a rheumatologist are taking csDMARDs to manage RA, while nearly one-third receive biologic treatment following csDMARD treatment. This is greater than UK-based statistics of 21% (84 200/40 000) of RA patients receiving biologic treatment [33].

Our data show there was a trend towards the highest treatment with biologics in the year 2014, which peaks and troughs for the subsequent years for non-anti-IL-6 bDMARDs. This can be explained by tocilizumab and sarilumab becoming available to treat RA in the UK in 2012 and 2017, respectively, and also changing to alternative treatment options due to lack of an adequate response [1]. However, tocilizumab and sarilumab have steady usage from 2017 to 2020.

Factors associated with earlier treatment with non-anti-IL-6 bDMARDs included increased disease duration. However, orthopaedic surgery and kidney disease were negatively associated with commencing non-anti-IL-6 bDMARDs, which highlights the impact of comorbidities on starting treatment.

For anti-IL-6 bDMARDs, a history of infections requiring hospital treatment was associated with an earlier start with the treatment. This is interesting since tocilizumab has often been associated with an increased risk of infection, yet in our findings from real-world data, tocilizumab/sarilumab showed a lower rate of post-treatment infections requiring hospital treatment compared with non-anti-IL-6 bDMARDs.

More than 23% of the bDMARD cohort switched or added biologic treatments, which was taken as a proxy for treatment failure/change, as evidenced by the need to add new drugs to the treatment regimen. The reason for treatment failure appeared to be related to more severe or poorly controlled disease, as suggested by the history of orthopaedic surgery and high steroid use. No factors were shown to be associated with patients who failed anti-IL-6 bDMARDs, however, this may be a result of fewer patients taking the drugs and thus could be statistically underpowered. Another potential explanation is the greater number of patients with hypertension, hyperlipidaemia and steroid use. Hyperlipidaemia is associated with anti-IL-6 bDMARDs and could lead to the higher rate of discontinuation.

### Strengths

This study reports on the treatment of nearly 5000 individuals in the UK using linked electronic health data from multiple sources with a confirmed diagnosis by a rheumatologist. The study is multicentre and links data from various sources for a sustained follow-up period.

The demographic characteristics of the cohort are comparable to recent work using data from the BSRBR-RA. For instance, our sample was 72% female and had a mean age of 62 years compared with the BSRBR-RA study, which was 76% female, with an average age of 56 years. However, the disease duration of 6.3 years in our study is half that of the BSRBR-RA study, which was 12.7 years [29].

### Limitations

The cohort had established and perhaps more severe disease, as they were under the care of a rheumatologist. As such, the data presented here may not be representative of early and/or less severe rheumatoid patients.

The absence of ESR and CRP results may be considered a shortcoming in this study. Disease activity scores were available but not well-populated, so they were excluded from this study.

We were potentially underpowered to detect associations of individuals who failed treatment with anti-IL-6 bDMARDs due to lower usage numbers in the data, so CIs of estimates will lack precision, e.g. there were only 19 individuals who had IL-6 bDMARD monotherapy and we were underpowered to detect associations of individuals who failed treatment with anti-IL-6 bDMARDs due to lower numbers. Therefore, in some cases where no significant factors were identified, this may be because we were underpowered to detect a difference, if one existed. Further study is needed with more data to assess factors associated with anti-IL-6 bDMARD failure.

No end dates are supplied for the medications prescribed. As such, treatment ‘failure’ is defined as a change in the listed medication, i.e. removal of the medication. This is an assumption and requires more thorough examination in future studies. Also, data for the reason for treatment change or failure are not available.

This study relies on routine data where there was an absence of quality-of-life measures, which could have been useful to assess patient outcomes.

## Conclusion

This study finds that as per National Institute for Health and Care Excellence guidelines, the majority of RA patients were treated with csDMARDS as the first line of treatment. One-third of the patients went on to receive biologic and tocilizumab or sarilumab treatment following csDMARD use, which is higher than UK estimates reported elsewhere [33]. Biologic treatment failure appears to be associated with poorly controlled disease, which highlights the necessity for more prompt treatment.

As this cohort is sourced from secondary care data, these are more severe patients with established disease, however, the treatment, care pathways and health outcomes as observed from these real-world data are useful to assess the treatment of RA in the UK.

## Supplementary Material

rkae140_Supplementary_Data

## Data Availability

The data underlying this article cannot be shared publicly due to the policies and procedures in place to protect data held in the SAIL Databank.
