# Supplementary material for: The pattern of anti-IL-6 versus non-anti-IL-6 biologic disease modifying anti-rheumatic drugs use in patients with rheumatoid arthritis in Wales, UK: a real-world study using electronic health records
Source: Rheumatol Adv Pract. 2024 Dec 14;9(1):rkae140. doi: 10.1093/rap/rkae140 (PMC11651880; doi:10.1093/rap/rkae140)
Supplement: rkae140_Supplementary_Data [file rkae140_supplementary_data.docx]

**Supplementary Table S1: SAIL Databank sources for cohort data**

|  | Rheumatology  clinic data | Primary care  Data) | Secondary care  data | Office National Statistics  Death Dataset |
| --- | --- | --- | --- | --- |
| Gender^α^ |  |  |  |  |
| Age^β^ |  |  |  |  |
| BMI^β^ |  |  |  |  |
| Smoker^α^ |  |  |  |  |
| Living in rural area^α^ |  |  |  |  |
| RA codes^α^ |  |  |  |  |
| DMARDs^α^ |  |  |  |  |
| Biologic agents^α^ |  |  |  |  |
| Tocilizumab/Sarilumab^α^ |  |  |  |  |
| Steroids^α^ |  |  |  |  |
| Hyperlipidaemia^α^ |  |  |  |  |
| Hypertension^α^ |  |  |  |  |
| Diabetes^α^ |  |  |  |  |
| Cardiovascular disease^α^ |  |  |  |  |
| Cancer^α^ |  |  |  |  |
| Infections^α^ |  |  |  |  |
| Orthopaedic surgery^α^ |  |  |  |  |
| Kidney disease^α^ |  |  |  |  |
| Date of death |  |  |  |  |

*Categorical variable ^α^; continuous variable ^β^*

**Supplementary Table S2: Definition of conditions primary care records using the READ code system**

| **Read Code** | **Description** | | |  |
| --- | --- | --- | --- | --- |
| **Cardiovascular disease** | | | |  |
| 14A3. | H/O: myocardial infarct <60 | | |  |
| 14A4. | H/O: myocardial infarct >60 | | |  |
| 14A5. | H/O: angina pectoris | | |  |
| 14A6. | H/O: heart failure | | |  |
| 14AH. | H/O: Myocardial infarction in last year | | |  |
| 14AJ. | H/O: Angina in last year | | |  |
| 14AM. | H/O: Heart failure in last year | | |  |
| 14AT. | History of myocardial infarction | | |  |
| 14AW. | H/O acute coronary syndrome | | |  |
| 14NB. | H/O: Peripheral vascular disease procedure | | |  |
| 1J60. | Suspected heart failure | | |  |
| 1O1.. | Heart failure confirmed | | |  |
| 21264 | Heart failure resolved | | |  |
| 323.. | ECG: myocardial infarction | | |  |
| 3232 | ECG: old myocardial infarction | | |  |
| 323Z. | ECG: myocardial infarct NOS | | |  |
| 388D. | New York Heart Assoc classification heart failure symptoms | | |  |
| 661M5 | Heart failure self-management plan agreed | | |  |
| 662p. | Heart failure 6 month review | | |  |
| 662T. | Congestive heart failure monitoring | | |  |
| 662W. | Heart failure annual review | | |  |
| 679W1 | Education about deteriorating heart failure | | |  |
| 679X. | Heart failure education | | |  |
| 67D4. | Heart failure information given to patient | | |  |
| 792.. | Coronary artery operations | | |  |
| 7920 | Saphenous vein graft replacement of coronary artery | | |  |
| 7921 | Other autograft replacement of coronary artery | | |  |
| 7922 | Allograft replacement of coronary artery | | |  |
| 7923 | Prosthetic replacement of coronary artery | | |  |
| 7924 | Revision of bypass for coronary artery | | |  |
| 79240 | Revision of bypass for one coronary artery | | |  |
| 79241 | Revision of bypass for two coronary arteries | | |  |
| 79242 | Revision of bypass for three coronary arteries | | |  |
| 79243 | Revision of bypass for four or more coronary arteries | | |  |
| 7924y | Other specified revision of bypass for coronary artery | | |  |
| 7924z | Revision of bypass for coronary artery NOS | | |  |
| 7925 | Connection of mammary artery to coronary artery | | |  |
| 79275 | Open angioplasty of coronary artery | | |  |
| 7928 | Transluminal balloon angioplasty of coronary artery | | |  |
| 79280 | Percut transluminal balloon angioplasty one coronary artery | | |  |
| 79281 | Percut translum balloon angioplasty mult coronary arteries | | |  |
| 79282 | Percut translum balloon angioplasty bypass graft coronary a | | |  |
| 79283 | Percut translum cutting balloon angioplasty coronary artery | | |  |
| 7928y | Transluminal balloon angioplasty of coronary artery OS | | |  |
| 7928z | Transluminal balloon angioplasty of coronary artery NOS | | |  |
| 79290 | Percutaneous transluminal laser coronary angioplasty | | |  |
| 79293 | Rotary blade coronary angioplasty | | |  |
| 79294 | Insertion of coronary artery stent | | |  |
| 79295 | Insertion of drug-eluting coronary artery stent | | |  |
| 792D. | Other bypass of coronary artery | | |  |
| 792Dy | Other specified other bypass of coronary artery | | |  |
| 792Dz | Other bypass of coronary artery NOS | | |  |
| 793G. | Perc translumin balloon angioplasty stenting coronary artery | | |  |
| 793Gy | OS perc translumina balloon angioplast stenting coronary art | | |  |
| 793Gz | Perc translum balloon angioplasty stenting coronary art NOS | | |  |
| 889A. | Diab mellit insulin-glucose infus acute myocardial infarct | | |  |
| 8CeC. | Preferred place of care for next exacerbation heart failure | | |  |
| 8CL3. | Heart failure care plan discussed with patient | | |  |
| 8CMK. | Has heart failure management plan | | |  |
| 8CMW8 | Heart failure clinical pathway | | |  |
| 8H2S. | Admit heart failure emergency | | |  |
| 8HBE. | Heart failure follow-up | | |  |
| 8HBJ. | Stroke / transient ischaemic attack referral | | |  |
| 8L40. | Coronary artery bypass graft operation planned | | |  |
| 8L41. | Coronary angioplasty planned | | |  |
| G1yz1 | Rheumatic left ventricular failure | | |  |
| G232. | Hypertensive heart&renal dis wth (congestive) heart failure | | |  |
| G3... | Ischaemic heart disease | | |  |
| G30.. | Acute myocardial infarction | | |  |
| G301. | Other specified anterior myocardial infarction | | |  |
| G301z | Anterior myocardial infarction NOS | | |  |
| G304. | Posterior myocardial infarction NOS | | |  |
| G305. | Lateral myocardial infarction NOS | | |  |
| G306. | True posterior myocardial infarction | | |  |
| G3071 | Acute non-ST segment elevation myocardial infarction | | |  |
| G308. | Inferior myocardial infarction NOS | | |  |
| G30B. | Acute posterolateral myocardial infarction | | |  |
| G30X. | Acute transmural myocardial infarction of unspecif site | | |  |
| G30X0 | Acute ST segment elevation myocardial infarction | | |  |
| G30y. | Other acute myocardial infarction | | |  |
| G30yz | Other acute myocardial infarction NOS | | |  |
| G30z. | Acute myocardial infarction NOS | | |  |
| G31.. | Other acute and subacute ischaemic heart disease | | |  |
| G310. | Postmyocardial infarction syndrome | | |  |
| G311. | Preinfarction syndrome | | |  |
| G3110 | Myocardial infarction aborted | | |  |
| G3111 | Unstable angina | | |  |
| G3113 | Refractory angina | | |  |
| G3115 | Acute coronary syndrome | | |  |
| G31y. | Other acute and subacute ischaemic heart disease | | |  |
| G31y0 | Acute coronary insufficiency | | |  |
| G31yz | Other acute and subacute ischaemic heart disease NOS | | |  |
| G32.. | Old myocardial infarction | | |  |
| G33.. | Angina pectoris | | |  |
| G331. | Prinzmetal's angina | | |  |
| G33z. | Angina pectoris NOS | | |  |
| G33z3 | Angina on effort | | |  |
| G33z5 | Post infarct angina | | |  |
| G33z7 | Stable angina | | |  |
| G33zz | Angina pectoris NOS | | |  |
| G34.. | Other chronic ischaemic heart disease | | |  |
| G340. | Coronary atherosclerosis | | |  |
| G34y. | Other specified chronic ischaemic heart disease | | |  |
| G34yz | Other specified chronic ischaemic heart disease NOS | | |  |
| G34z. | Other chronic ischaemic heart disease NOS | | |  |
| G35.. | Subsequent myocardial infarction | | |  |
| G350. | Subsequent myocardial infarction of anterior wall | | |  |
| G351. | Subsequent myocardial infarction of inferior wall | | |  |
| G353. | Subsequent myocardial infarction of other sites | | |  |
| G35X. | Subsequent myocardial infarction of unspecified site | | |  |
| G36.. | Certain current complication follow acute myocardial infarct | | |  |
| G38.. | Postoperative myocardial infarction | | |  |
| G380. | Postoperative transmural myocardial infarction anterior wall | | |  |
| G381. | Postoperative transmural myocardial infarction inferior wall | | |  |
| G383. | Postoperative transmural myocardial infarction unspec site | | |  |
| G384. | Postoperative subendocardial myocardial infarction | | |  |
| G38z. | Postoperative myocardial infarction, unspecified | | |  |
| G3y.. | Other specified ischaemic heart disease | | |  |
| G3z.. | Ischaemic heart disease NOS | | |  |
| G58.. | Heart failure | | |  |
| G580. | Congestive heart failure | | |  |
| G5800 | Acute congestive heart failure | | |  |
| G5801 | Chronic congestive heart failure | | |  |
| G5804 | Congestive heart failure due to valvular disease | | |  |
| G581. | Left ventricular failure | | |  |
| G5810 | Acute left ventricular failure | | |  |
| G582. | Acute heart failure | | |  |
| G583. | Heart failure with normal ejection fraction | | |  |
| G584. | Right ventricular failure | | |  |
| G58z. | Heart failure NOS | | |  |
| G5y4z | Post cardiac operation heart failure NOS | | |  |
| G670. | Cerebral atherosclerosis | | |  |
| G677. | Occlusion/stenosis cerebral arts not result cerebral infarct | | |  |
| G70.. | Atherosclerosis | | |  |
| G700. | Aortic atherosclerosis | | |  |
| G701. | Renal artery atherosclerosis | | |  |
| G70y0 | Carotid artery atherosclerosis | | |  |
| Gyu3. | [X]Ischaemic heart diseases | | |  |
| Gyu30 | [X]Other forms of angina pectoris | | |  |
| Gyu32 | [X]Other forms of acute ischaemic heart disease | | |  |
| Gyu33 | [X]Other forms of chronic ischaemic heart disease | | |  |
| Gyu34 | [X]Acute transmural myocardial infarction of unspecif site | | |  |
| Gyu36 | [X]Subsequent myocardial infarction of unspecified site | | |  |
| ZV457 | [V]Presence of aortocoronary bypass graft | | |  |
| ZV458 | [V]Presence of coronary angioplasty implant and graft | | |  |
| ZV45K | [V]Presence of coronary artery bypass graft | | |  |
| **Diabetes** |  | | |  |
| C1001 | Diabetes mellitus, adult onset, no mention of complication | | |  |
| C1011 | Diabetes mellitus, adult onset, with ketoacidosis | | |  |
| C1021 | Diabetes mellitus, adult onset, with hyperosmolar coma | | |  |
| C1031 | Diabetes mellitus, adult onset, with ketoacidotic coma | | |  |
| C1041 | Diabetes mellitus, adult onset, with renal manifestation | | |  |
| C1051 | Diabetes mellitus, adult onset, + ophthalmic manifestation | | |  |
| C1061 | Diabetes mellitus, adult onset, + neurological manifestation | | |  |
| C1071 | Diabetes mellitus, adult, + peripheral circulatory disorder | | |  |
| C1072 | Diabetes mellitus, adult with gangrene | | |  |
| C1074 | NIDDM with peripheral circulatory disorder | | |  |
| C109. | Non-insulin dependent diabetes mellitus | | |  |
| C1090 | Non-insulin-dependent diabetes mellitus with renal comps | | |  |
| C1091 | Non-insulin-dependent diabetes mellitus with ophthalm comps | | |  |
| C1092 | Non-insulin-dependent diabetes mellitus with neuro comps | | |  |
| C1093 | Non-insulin-dependent diabetes mellitus with multiple comps | | |  |
| C1094 | Non-insulin dependent diabetes mellitus with ulcer | | |  |
| C1095 | Non-insulin dependent diabetes mellitus with gangrene | | |  |
| C1096 | Non-insulin-dependent diabetes mellitus with retinopathy | | |  |
| C1097 | Non-insulin dependent diabetes mellitus - poor control | | |  |
| C1099 | Non-insulin-dependent diabetes mellitus without complication | | |  |
| C109A | Non-insulin dependent diabetes mellitus with mononeuropathy | | |  |
| C109B | Non-insulin dependent diabetes mellitus with polyneuropathy | | |  |
| C109C | Non-insulin dependent diabetes mellitus with nephropathy | | |  |
| C109D | Non-insulin dependent diabetes mellitus with hypoglyca coma | | |  |
| C109E | Non-insulin depend diabetes mellitus with diabetic cataract | | |  |
| C109F | Non-insulin-dependent d m with peripheral angiopath | | |  |
| C109G | Non-insulin dependent diabetes mellitus with arthropathy | | |  |
| C109H | Non-insulin dependent d m with neuropathic arthropathy | | |  |
| C109J | Insulin treated Type 2 diabetes mellitus | | |  |
| C109K | Hyperosmolar non-ketotic state in type 2 diabetes mellitus | | |  |
| C10F. | Type 2 diabetes mellitus | | |  |
| C10F0 | Type 2 diabetes mellitus with renal complications | | |  |
| C10F1 | Type 2 diabetes mellitus with ophthalmic complications | | |  |
| C10F2 | Type 2 diabetes mellitus with neurological complications | | |  |
| C10F3 | Type 2 diabetes mellitus with multiple complications | | |  |
| C10F4 | Type 2 diabetes mellitus with ulcer | | |  |
| C10F5 | Type 2 diabetes mellitus with gangrene | | |  |
| C10F6 | Type 2 diabetes mellitus with retinopathy | | |  |
| C10F7 | Type 2 diabetes mellitus - poor control | | |  |
| C10F9 | Type 2 diabetes mellitus without complication | | |  |
| C10FA | Type 2 diabetes mellitus with mononeuropathy | | |  |
| C10FB | Type 2 diabetes mellitus with polyneuropathy | | |  |
| C10FC | Type 2 diabetes mellitus with nephropathy | | |  |
| C10FD | Type 2 diabetes mellitus with hypoglycaemic coma | | |  |
| C10FE | Type 2 diabetes mellitus with diabetic cataract | | |  |
| C10FF | Type 2 diabetes mellitus with peripheral angiopathy | | |  |
| C10FG | Type 2 diabetes mellitus with arthropathy | | |  |
| C10FH | Type 2 diabetes mellitus with neuropathic arthropathy | | |  |
| C10FJ | Insulin treated Type 2 diabetes mellitus | | |  |
| C10FK | Hyperosmolar non-ketotic state in type 2 diabetes mellitus | | |  |
| C10FL | Type 2 diabetes mellitus with persistent proteinuria | | |  |
| C10FM | Type 2 diabetes mellitus with persistent microalbuminuria | | |  |
| C10FN | Type 2 diabetes mellitus with ketoacidosis | | |  |
| C10FQ | Type 2 diabetes mellitus with exudative maculopathy | | |  |
| C10FR | Type 2 diabetes mellitus with gastroparesis | | |  |
| C10y1 | Diabetes mellitus, adult, + other speciﬁed manifestation | | |  |
| C10z1 | Diabetes mellitus, adult onset, + unspeciﬁed complication | | |  |
| L1806 | Pre-existing diabetes mellitus, non-insulin-dependent | | |  |
| **Hypertension** | |  |  |  |
| G200. | | Malignant essential hypertens. |  |  |
| G220. | | Malignant hypertens.renal dis. |  |  |
| G2100 | | Malig.hypert.heart dis.-no CCF |  |  |
| G210z | | Malig.hypertens.heart dis. NOS |  |  |
| G2111 | | Benign hypert.heart dis-+ CCF |  |  |
| G2400 | | Second.malig.renovasc.hypert. |  |  |
| G240z | | Secondary malign.hypertens.NOS |  |  |
| G201. | | Benign essential hypertension |  |  |
| G221. | | Benign hypertensive renal dis. |  |  |
| G21z0 | | Hypertens.heart dis.NOS-no CCF |  |  |
| G24z0 | | Secondary renovasc.hypert. NOS |  |  |
| G2... | | Hypertensive disease |  |  |
| G20.. | | Essential hypertension |  |  |
| G22.. | | Hypertensive renal disease |  |  |
| G26.. | | Severe hypertensin (NICE 2011) |  |  |
| G27.. | | Hypertnsn resistnt to drg ther |  |  |
| G2y.. | | Hypertensive disease OS |  |  |
| G20z. | | Essential hypertension NOS |  |  |
| G22z. | | Hypertensive renal disease NOS |  |  |
| G203. | | Diastolic hypertension |  |  |
| G232. | | Hypert ht&ren d+(congs)ht fail |  |  |
| G234. | | Hyp ht&ren d+both(con)h&r fail |  |  |
| G244. | | Hypertens 2ndry endocrin disor |  |  |
| G202. | | Systolic hypertension |  |  |
| G21z1 | | Hypertens.heart dis.NOS- + CCF |  |  |
| G222. | | Hypertens renal dis+renal fail |  |  |
| G233. | | Hypertn hrt&ren dis+renal fail |  |  |
| G24z1 | | Hypertension secondary to drug |  |  |
| G21zz | | Hypertensive heart disease NOS |  |  |
| G24zz | | Secondary hypertension NOS |  |  |
| G21.. | | Hypertensive heart disease |  |  |
| G2101 | | Malig.hypert.heart dis.-+ CCF |  |  |
| G2110 | | Benign hypert.heart dis-no CCF |  |  |
| G211z | | Benign hypertens.heart dis.NOS |  |  |
| G23.. | | Hypertensive heart+renal dis. |  |  |
| G24.. | | Secondary hypertension |  |  |
| G2410 | | Second.benign renovasc.hypert. |  |  |
| G241z | | Secondary benign hypertens.NOS |  |  |
| G25.. | | Stge 1 hypertensin (NICE 2011) |  |  |
| G28.. | | Stge 2 hypertensin (NICE 2011) |  |  |
| G2z.. | | Hypertensive disease NOS |  |  |
| G211. | | Benign hypertensive heart dis. |  |  |
| G231. | | Benign hypert.heart+renal dis. |  |  |
| G241. | | Secondary benign hypertension |  |  |
| G251. | | Stage 1 hyp wi ev end org dmge |  |  |
| G21z. | | Hypertensive heart disease NOS |  |  |
| G23z. | | Hypertens.heart+renal dis.NOS |  |  |
| G24z. | | Secondary hypertension NOS |  |  |
| G210. | | Malignant hypertens.heart dis. |  |  |
| G230. | | Malig.hypert.heart+renal dis. |  |  |
| G240. | | Secondary malignant hypertens. |  |  |
| G250. | | Stage 1 hyp wo ev end org dmge |  |  |
| **Hyperlipidaemia** | |  | | |
| C324. | | Hyperlipidaemia NOS | | |
| C322. | | Mixed hyperlipidaemia | | |
| C3201 | | Hyperbetalipoproteinaemia | | |
| C3203 | | LDL hyperlipoproteinaemia | | |
| C320. | | Pure hypercholesterolaemia | | |
| C320y | | Pure hypercholesterolaemia OS | | |
| C3200 | | Familial hypercholesterolaemia | | |
| C320z | | Pure hypercholesterolaemia NOS | | |
| C3202 | | Hyperlipidaemia, group A | | |
| C3206 | | Polygenic hypercholesterolemia | | |
| C3205 | | Fam defect apolipoprot B-100 | | |
| C3204 | | Fredrickson type IIa lipidaem | | |
| C328. | | Dyslipidaemia | | |
| C321. | | Pure hyperglyceridaemia | | |
| C3210 | | Hypertriglyceridaemia | | |
| **Rheumatoid arthritis** | | | | |
| G5y8. | | Rheumatoid myocarditis | | |
| G5yA. | | Rheumatoid carditis | | |
| N040. | | Rheumatoid arthritis | | |
| N0400 | | Rheumatoid arthritis of cervical spine | | |
| N0401 | | Other rheumatoid arthritis of spine | | |
| N0402 | | Rheumatoid arthritis of shoulder | | |
| N0404 | | Rheumatoid arthritis of acromioclavicular joint | | |
| N0405 | | Rheumatoid arthritis of elbow | | |
| N0406 | | Rheumatoid arthritis of distal radio-ulnar joint | | |
| N0407 | | Rheumatoid arthritis of wrist | | |
| N0408 | | Rheumatoid arthritis of MCP joint | | |
| N0409 | | Rheumatoid arthritis of PIP joint of finger | | |
| N040A | | Rheumatoid arthritis of DIP joint of finger | | |
| N040B | | Rheumatoid arthritis of hip | | |
| N040C | | Rheumatoid arthritis of sacro-iliac joint | | |
| N040D | | Rheumatoid arthritis of knee | | |
| N040F | | Rheumatoid arthritis of ankle | | |
| N040G | | Rheumatoid arthritis of subtalar joint | | |
| N040H | | Rheumatoid arthritis of talonavicular joint | | |
| N040J | | Rheumatoid arthritis of other tarsal joint | | |
| N040K | | Rheumatoid arthritis of 1st MTP joint | | |
| N040L | | Rheumatoid arthritis of lesser MTP joint | | |
| N040N | | Rheumatoid vasculitis | | |
| N040P | | Seronegative rheumatoid arthritis | | |
| N040R | | Rheumatoid nodule | | |
| N040S | | Rheumatoid arthritis - multiple joint | | |
| N040T | | Flare of rheumatoid arthritis | | |
| N041. | | Felty's syndrome | | |
| N042. | | Other rheumatoid arthropathy + visceral/systemic involvement | | |
| N0421 | | Rheumatoid lung disease | | |
| N0422 | | Rheumatoid nodule | | |
| N042z | | Rheumatoid arthropathy + visceral/systemic involvement NOS | | |
| N047. | | Seropositive errosive rheumatoid arthritis | | |
| N04X. | | Seropositive rheumatoid arthritis, unspecified | | |
| N04y0 | | Rheumatoid lung | | |
| N04y2 | | Adult-onset Still's disease | | |
| Nyu10 | | [X]Rheumatoid arthritis+involvement/other organs or systems | | |
| Nyu11 | | [X]Other seropositive rheumatoid arthritis | | |
| Nyu12 | | [X]Other specified rheumatoid arthritis | | |
| Nyu1G | | [X]Seropositive rheumatoid arthritis, unspecified | | |

**Supplementary Table S3: Definition of drugs from primary care records using the READ code system**

| **Read Code** | **Description** | | | |  |  |
| --- | --- | --- | --- | --- | --- | --- |
| **DMARDs** |  | | | |  |  |
| ej26. | Chloroquine phosphate 250mg tablets | | | |  |  |
| ej25. | Nivaquine 272.5mg(200mg base)/5ml Injection (Aventis Pharma) | | | |  |  |
| ejC.. | Chloroquine phosphate 250mg tablets and Proguanil 100mg tablets | | | |  |  |
| j54z. | Hydroxychloroquine 200mg tablets | | | |  |  |
| ej24. | Nivaquine 68mg/5ml Oral solution (Aventis Pharma) | | | |  |  |
| ej23. | Nivaquine 200mg Tablet (Aventis Pharma) | | | |  |  |
| ej21. | Avloclor 250mg tablets (AstraZeneca UK Ltd) | | | |  |  |
| ej31. | Plaquenil 200mg tablets (Sanofi) | | | |  |  |
| j541. | Plaquenil 200mg tablets (Sanofi) | | | |  |  |
| ej2w. | Chloroquine phosphate 80mg/5ml oral solution | | | |  |  |
| ej22. | Malarivon 80mg/5ml syrup (Wallace Manufacturing Chemists Ltd) | | | |  |  |
| j542. | Quinoric 200mg tablets (Bristol Laboratories Ltd) | | | |  |  |
| h871. | Adalimumab 40mg injection | | | |  |  |
| h873. | Adalimumab 40mg injection | | | |  |  |
| h89w. | Enbrel 25mg powder and solvent for solution for injection vials (Pfizer Ltd) | | | |  |  |
| h892. | Etanercept 25mg powder and solvent for solution for injection vials | | | |  |  |
| h8Bz. | Infliximab 100mg powder for solution for infusion vials | | | |  |  |
| h89z. | Enbrel 50mg powder and solvent for solution for injection vials (Wyeth Pharmaceuticals) | | | |  |  |
| h8B1. | Remicade 100mg powder for solution for infusion vials (Merck Sharp & Dohme Ltd) | | | |  |  |
| h872. | Humira 40mg Injection (Abbott Laboratories Ltd) | | | |  |  |
| h874. | Humira 40mg Injection (Abbott Laboratories Ltd) | | | |  |  |
| h891. | Etanercept 50mg powder and solvent for solution for injection vials | | | |  |  |
| h893. | Etanercept 50mg injection solution | | | |  |  |
| h895. | Etanercept 50mg injection solution | | | |  |  |
| h89v. | Enbrel 25mg/0.5ml solution for injection pre-filled syringes (Pfizer Ltd) | | | |  |  |
| h894. | Etanercept 25mg/0.5ml solution for injection pre-filled syringes | | | |  |  |
| h89u. | Enbrel 50mg Solution for injection (Pfizer Consumer Healthcare Ltd) | | | |  |  |
| h89x. | Enbrel 50mg Solution for injection (Pfizer Consumer Healthcare Ltd) | | | |  |  |
| h89y. | Enbrel Paediatric 25mg powder and solvent for solution for injection vials (Pfizer Ltd) | | | |  |  |
| h8G2. | Cimzia 200mg/1ml solution for injection pre-filled syringes (UCB Pharma Ltd) | | | |  |  |
| h8G1. | Certolizumab pegol 200mg/1ml solution for injection pre-filled syringes | | | |  |  |
| h71z. | Azathioprine 50mg powder for solution for injection vials | | | |  |  |
| h71y. | Azathioprine 25mg tablets | | | |  |  |
| h71x. | Azathioprine 50mg tablets | | | |  |  |
| h712. | Imuran 25mg Tablet (Wellcome Medical Division) | | | |  |  |
| h713. | Imuran 50mg Tablet (Wellcome Medical Division) | | | |  |  |
| h711. | Azamune 50mg Tablet (Penn Pharmaceuticals Ltd) | | | |  |  |
| h718. | Azathioprine 10mg tablets | | | |  |  |
| h714. | Imuran 50mg powder for solution for injection vials (Aspen Pharma Trading Ltd) | | | |  |  |
| h717. | Oprisine 50mg Tablet (Opus Pharmaceuticals Ltd) | | | |  |  |
| h715. | Immunoprin 50mg tablets (Ashbourne Pharmaceuticals Ltd) | | | |  |  |
| h716. | Berkaprine 50mg Tablet (Rorer Pharmaceuticals Ltd) | | | |  |  |
| h719. | Imuran 10mg Tablet (Wellcome Medical Division) | | | |  |  |
| hh11. | Rituximab 100mg/10ml solution for infusion vials | | | |  |  |
| hh12. | Rituximab 500mg/50ml solution for infusion vials | | | |  |  |
| h14.. | Cyclophosphamide | | | |  |  |
| h146. | Endoxana 10mg tablet | | | |  |  |
| h141. | Cyclophosphamide 50mg tablet | | | |  |  |
| h147. | Endoxana 50mg tablet | | | |  |  |
| h142. | Cyclophosphamide 100mg injection (pdr for recon) | | | |  |  |
| h148. | Endoxana 100mg injection (pdr for recon) | | | |  |  |
| h143. | Cyclophosphamide 200mg injection (pdr for recon) | | | |  |  |
| h14A. | Cyclophos 200mg injection (pdr for recon) | | | |  |  |
| h149. | Endoxana 200mg injection (pdr for recon) | | | |  |  |
| h144. | Cyclophosphamide 500mg injection (pdr for recon) | | | |  |  |
| h14B. | Cyclophos 500mg injection (pdr for recon) | | | |  |  |
| h145. | Cyclophosphamide 1g injection (pdr for recon) | | | |  |  |
| h14C. | Cyclophos 1g injection (pdr for recon) | | | |  |  |
| x00Nb | Cyclophosphamide 1g/50mL infusion | | | |  |  |
| x00Na | Cyclophosphamide 2g/100mL infusion | | | |  |  |
| x00NZ | Cyclophosphamide 4g/200mL infusion | | | |  |  |
| h82A. | Neoral 25mg capsules (Novartis Pharmaceuticals UK Ltd) | | | |  |  |
| h82C. | Neoral 100mg capsules (Novartis Pharmaceuticals UK Ltd) | | | |  |  |
| h82x. | Ciclosporin 100mg/ml oral solution sugar free | | | |  |  |
| h82D. | Neoral 100mg/ml oral solution (Novartis Pharmaceuticals UK Ltd) | | | |  |  |
| h829. | Ciclosporin 50mg capsules | | | |  |  |
| h826. | Ciclosporin 25mg capsules | | | |  |  |
| h827. | Ciclosporin 100mg capsules | | | |  |  |
| h824. | Sandimmun 25mg capsules (Novartis Pharmaceuticals UK Ltd) | | | |  |  |
| h82B. | Neoral 50mg capsules (Novartis Pharmaceuticals UK Ltd) | | | |  |  |
| h821. | Sandimmun 100mg/ml oral solution (Novartis Pharmaceuticals UK Ltd) | | | |  |  |
| h825. | Sandimmun 100mg capsules (Novartis Pharmaceuticals UK Ltd) | | | |  |  |
| h828. | Sandimmun 50mg capsules (Novartis Pharmaceuticals UK Ltd) | | | |  |  |
| h82E. | Ciclosporin 10mg capsules | | | |  |  |
| h82F. | Neoral 10mg capsules (Novartis Pharmaceuticals UK Ltd) | | | |  |  |
| h822. | Sandimmun 50mg/ml Concentrate for solution for infusion (Novartis Pharmaceuticals UK Ltd) | | | |  |  |
| h82y. | Ciclosporin 50mg/1ml solution for infusion ampoules | | | |  |  |
| h82I. | Deximune 50mg capsules (Dexcel-Pharma Ltd) | | | |  |  |
| h82J. | Deximune 100mg capsules (Dexcel-Pharma Ltd) | | | |  |  |
| h82H. | Deximune 25mg capsules (Dexcel-Pharma Ltd) | | | |  |  |
| h82z. | Ciclosporin 250mg/5ml solution for infusion ampoules | | | |  |  |
| h82.. | Ciclosporin product | | | |  |  |
| h82G. | Sangcya 100mg/mL oral solution | | | |  |  |
| h823. | Sandimmun 250mg/5mL oily infusion concentrate | | | |  |  |
| h82K. | CAPIMUNE 25mg capsules | | | |  |  |
| h82L. | CAPIMUNE 50mg capsules | | | |  |  |
| h82M. | CAPIMUNE 100mg capsules | | | |  |  |
| h82N. | CAPSORIN 25mg capsules | | | |  |  |
| h82O. | CAPSORIN 50mg capsules | | | |  |  |
| h82P. | CAPSORIN 100mg capsules | | | |  |  |
| j513. | Myocrisin 10mg/0.5ml solution for injection ampoules (Sanofi) | | | |  |  |
| j515. | Myocrisin 50mg/0.5ml solution for injection ampoules (Sanofi) | | | |  |  |
| j514. | Myocrisin 20mg/0.5ml solution for injection ampoules (Sanofi) | | | |  |  |
| j51z. | Sodium aurothiomalate 50mg/0.5ml solution for injection ampoules | | | |  |  |
| j51x. | Sodium aurothiomalate 10mg/0.5ml solution for injection ampoules | | | |  |  |
| j51y. | Sodium aurothiomalate 20mg/0.5ml solution for injection ampoules | | | |  |  |
| x01J9 | Hydroxychloroquine | | | |  |  |
| ej3.. | Hydroxychloroquine sulphate [anti malarial] | | | |  |  |
| j54.. | Hydroxychloroquine sulphate [anti- rheumatic] | | | |  |  |
| h862. | Kineret 100mg/0.67ml solution for injection pre-filled syringes (Swedish Orphan Biovitrum Ltd) | | | |  |  |
| h861. | Anakinra 100mg/0.67ml solution for injection pre-filled syringes | | | |  |  |
| h8Fz. | Tocilizumab 80mg/4ml solution for infusion vials | | | |  |  |
| h8Fy. | Tocilizumab 200mg/10ml solution for infusion vials | | | |  |  |
| j59z. | Leflunomide 100mg tablets | | | |  |  |
| j59x. | Leflunomide 10mg tablets | | | |  |  |
| j59y. | Leflunomide 20mg tablets | | | |  |  |
| j591. | Arava 10mg tablets (Sanofi) | | | |  |  |
| j592. | Arava 20mg tablets (Sanofi) | | | |  |  |
| j593. | Arava 100mg tablets (Sanofi) | | | |  |  |
| h34.. | METHOTREXATE | | | |  |  |
| h341. | METHOTREXATE 2.5mg tablets | | | |  |  |
| h342. | METHOTREXATE 10mg tablets | | | |  |  |
| h343. | METHOTREXATE 2.5mg/1mL solution for injection | | | |  |  |
| h344. | METHOTREXATE 5mg/2mL solution for injection | | | |  |  |
| h345. | METHOTREXATE 25mg/1mL solution for injection | | | |  |  |
| h346. | METHOTREXATE 50mg/2mL solution for injection | | | |  |  |
| h347. | METHOTREXATE 100mg/4mL injection solution | | | |  |  |
| h348. | METHOTREXATE 200mg/8mL solution for injection | | | |  |  |
| h349. | METHOTREXATE 500mg/20mL solution for injection | | | |  |  |
| h34A. | METHOTREXATE 15mg/1.5mL solution for injection prefilled syringe | | | |  |  |
| h34B. | METHOTREXATE 20mg/2mL solution for injection prefilled syringe | | | |  |  |
| h34C. | METHOTREXATE 25mg/2.5mL solution for injection prefilled syringe | | | |  |  |
| h34D. | METOJECT 7.5mg/0.75mL solution for injection prefilled syringe | | | |  |  |
| h34E. | METOJECT 10mg/1mL solution for injection prefilled syringe | | | |  |  |
| h34F. | METOJECT 15mg/1.5mL solution for injection prefilled syringe | | | |  |  |
| h34G. | METOJECT 20mg/2mL solution for injection prefilled syringe | | | |  |  |
| h34H. | METOJECT 25mg/2.5mL solution for injection prefilled syringe | | | |  |  |
| h34i. | EMTEXATE 1g/40mL solution for injection | | | |  |  |
| h34j. | EMTEXATE 5g/200mL solution for injection | | | |  |  |
| h34k. | EMTEXATE 1g/10mL solution for injection | | | |  |  |
| h34L. | METOJECT 7.5mg/0.15mL solution for injection pfs | | | |  |  |
| h34M. | METHOTREXATE 7.5mg/0.15mL solution for injection pfs | | | |  |  |
| h34N. | METOJECT 10mg/0.2mL solution for injection prefilled syringe | | | |  |  |
| h34O. | METHOTREXATE 10mg/0.2mL solution for injection pfs | | | |  |  |
| h34P. | METOJECT 15mg/0.3mL solution for injection prefilled syringe | | | |  |  |
| h34Q. | METHOTREXATE 15mg/0.3mL solution for injection pfs | | | |  |  |
| h34R. | METOJECT 20mg/0.4mL solution for injection prefilled syringe | | | |  |  |
| h34S. | METHOTREXATE 20mg/0.4mL solution for injection pfs | | | |  |  |
| h34T. | METOJECT 25mg/0.5mL solution for injection prefilled syringe | | | |  |  |
| h34U. | METHOTREXATE 25mg/0.5mL solution for injection pfs | | | |  |  |
| h34V. | METOJECT 30mg/0.6mL solution for injection prefilled syringe | | | |  |  |
| h34W. | METHOTREXATE 30mg/0.6mL solution for injection pfs | | | |  |  |
| h34X. | EBETREX 7.5mg/0.75mL soln for injection prefilled syringe | | | |  |  |
| h34Y. | EBETREX 10mg/1mL solution for injection prefilled syringe | | | |  |  |
| h34Z. | EBETREX 15mg/1.5mL solution for injection prefilled syringe | | | |  |  |
| h3G1. | EBETREX 20mg/1mL solution for injection prefilled syringe | | | |  |  |
| h3G2. | EBETREX 25mg/1.25mL solution for injection prefilled syringe | | | |  |  |
| h3G3. | EBETREX 30mg/1.5mL solution for injection prefilled syringe | | | |  |  |
| h3G4. | METHOTREXATE 20mg/1mL solution for injection p/f syringe | | | |  |  |
| h3G5. | METHOTREXATE 25mg/1.25mL solution for injection p/f syringe | | | |  |  |
| h3G6. | METHOTREXATE 30mg/1.5mL solution for injection p/f syringe | | | |  |  |
| h3G7. | METOJECT 12.5mg/0.25mL soln for injection prefilled syringe | | | |  |  |
| h3G8. | METHOTREXATE 12.5mg/0.25mL solution for injection pfs | | | |  |  |
| h3G9. | METOJECT 17.5mg/0.35mL soln for injection prefilled syringe | | | |  |  |
| h3GA. | METHOTREXATE 17.5mg/0.35mL solution for injection pfs | | | |  |  |
| h3GB. | METOJECT 22.5mg/0.45mL soln for injection prefilled syringe | | | |  |  |
| h3GC. | METHOTREXATE 22.5mg/0.45mL solution for injection pfs | | | |  |  |
| h3GD. | METOJECT 27.5mg/0.55mL soln for injection prefilled syringe | | | |  |  |
| h3GE. | METHOTREXATE 27.5mg/0.55mL solution for injection pfs | | | |  |  |
| j561. | Auranofin 3mg tablets | | | |  |  |
| j562. | Ridaura Tiltab 3mg tablets (Astellas Pharma Ltd) | | | |  |  |
| j521. | Penicillamine 50mg tablets | | | |  |  |
| j523. | Penicillamine 250mg tablets | | | |  |  |
| j522. | Penicillamine 125mg tablets | | | |  |  |
| j525. | Distamine 125mg tablets (Alliance Pharmaceuticals Ltd) | | | |  |  |
| j526. | Distamine 250mg tablets (Alliance Pharmaceuticals Ltd) | | | |  |  |
| j524. | Distamine 50mg Tablet (Alliance Pharmaceuticals Ltd) | | | |  |  |
| j528. | Pendramine 250mg Tablet (Viatris Pharmaceuticals Ltd) | | | |  |  |
| j527. | Pendramine 125mg Tablet (Viatris Pharmaceuticals Ltd) | | | |  |  |
| aa6z. | Sulfasalazine 3g/100ml enema | | | |  |  |
| aa64. | Salazopyrin 3g/100ml Enema (Pharmacia Ltd) | | | |  |  |
| aa62. | Salazopyrin EN-Tabs 500mg (Pfizer Ltd) | | | |  |  |
| j551. | Salazopyrin EN-Tabs 500mg (Pfizer Ltd) | | | |  |  |
| aa6v. | Sulfasalazine 500mg gastro-resistant tablets | | | |  |  |
| aa61. | Salazopyrin 500mg Tablet (Pharmacia Ltd) | | | |  |  |
| aa6y. | Sulfasalazine 500mg tablet | | | |  |  |
| j55z. | Sulfasalazine 500mg suppositories | | | |  |  |
| aa63. | Salazopyrin 500mg Suppository (Pharmacia Ltd) | | | |  |  |
| aa6u. | Sulfasalazine 250mg/5ml oral solution | | | |  |  |
| aa65. | Salazopyrin 250mg/5ml oral suspension (Pfizer Ltd) | | | |  |  |
| aa66. | Sulfasalazine 500mg gastro-resistant tablets (Actavis UK Ltd) | | | |  |  |
| j552. | Sulazine EC 500mg tablets (Genesis Pharmaceuticals Ltd) | | | |  |  |
| **Steroids** | | |  |  |  | |
| fe43. | | | *HYDROCORTISTAB 20mg tablets | | | |
| fe93. | | | *DEFLAZACORT 30mg tablets | | | |
| fe3A. | | | DEXSOL 2mg/5mL oral solution | | | |
| fe31. | | | DEXAMETHASONE 500micrograms tablets | | | |
| fe6k. | | | PREDNISOLONE 50mg tablets | | | |
| fe37. | | | *ORADEXON 2mg tablets | | | |
| fe3r. | | | DEXAMETHASONE 500micrograms/5mL solution | | | |
| fe66. | | | DELTACORTRIL ENTERIC 5mg tablets | | | |
| fe6a. | | | *DELTASTAB 5mg tablets | | | |
| fe6h. | | | PREDNISOLONE 2.5mg e/c tablets | | | |
| fe64. | | | *DELTA-PHORICOL 5mg tablets | | | |
| fe24. | | | *CORTISTAB 5mg tablets | | | |
| fe95. | | | *DEFLAZACORT 1mg tablets | | | |
| fe5p. | | | METHYLPREDNISOLONE 16mg tablets | | | |
| fe6e. | | | PRECORTISYL FORTE 25mg tablets | | | |
| fe94. | | | *CALCORT 30mg tablets | | | |
| fe21. | | | *CORTISONE 5mg tablets | | | |
| fe1y. | | | BETAMETHASONE 500microgram tablets | | | |
| fe1x. | | | BETAMETHASONE 500micrograms soluble tablets | | | |
| fe32. | | | DEXAMETHASONE 2mg tablets | | | |
| fe3u. | | | DEXAMETHASONE 2mg/5mL liquid | | | |
| fe5o. | | | METHYLPREDNISOLONE 4mg tablets | | | |
| fe65. | | | DELTACORTRIL ENTERIC 2.5mg tablets | | | |
| fe6d. | | | *PRECORTISYL 5mg tablets | | | |
| fe22. | | | CORTISONE 25mg tablets | | | |
| fb11. | | | FLORINEF 100micrograms tablets | | | |
| fe25. | | | *CORTISTAB 25mg tablets | | | |
| fe6j. | | | PREDNISOLONE 5mg soluble tablets | | | |
| fe68. | | | *DELTALONE 5mg tablets | | | |
| fe96. | | | *CALCORT 1mg tablets | | | |
| fe41. | | | HYDROCORTISONE 10mg tablets | | | |
| fe69. | | | *DELTASTAB 1mg tablets | | | |
| fe53. | | | MEDRONE 16mg tablets | | | |
| fe5m. | | | METHYLPREDNISOLONE 100mg tablets | | | |
| fe6i. | | | PREDNISOLONE 5mg e/c tablets | | | |
| fe42. | | | HYDROCORTISONE 20mg tablets | | | |
| fe11. | | | BETNELAN 500micrograms tablets | | | |
| fe5n. | | | METHYLPREDNISOLONE 2mg tablets | | | |
| fe23. | | | *CORTELAN 25mg tablets | | | |
| fe12. | | | BETNESOL 500micrograms tablets | | | |
| fe6z. | | | PREDNISOLONE 25mg tablets | | | |
| fe9.. | | | DEFLAZACORT | | | |
| fe2.. | | | CORTISONE ACETATE | | | |
| fe45. | | | *HYDROCORTONE 20mg tablets | | | |
| fe6v. | | | *PREDNISOLONE 2.5mg tablets | | | |
| fe3s. | | | DEXAMETHASONE 2mg/5mL sugar free solution | | | |
| fe67. | | | *DELTALONE 1mg tablets | | | |
| fe92. | | | CALCORT 6mg tablets | | | |
| fe36. | | | *ORADEXON 500microgram tablets | | | |
| fe44. | | | *HYDROCORTONE 10mg tablets | | | |
| fe62. | | | PREDNISOLONE 5mg tablets | | | |
| fe5f. | | | MEDRONE 100mg tablets | | | |
| fe33. | | | DECADRON 500micrograms tablets | | | |
| fe26. | | | *CORTISYL 25mg tablets | | | |
| fe6c. | | | *PRECORTISYL 1mg tablets | | | |
| fb1.. | | | FLUDROCORTISONE ACETATE | | | |
| fb1z. | | | FLUDROCORTISONE ACET 100microgram tablets | | | |
| fe6f. | | | *PREDNESOL 5mg tablets | | | |
| fe6g. | | | *SINTISONE 5mg tablets | | | |
| fe52. | | | MEDRONE 4mg tablets | | | |
| fe91. | | | DEFLAZACORT 6mg tablets | | | |
| fe51. | | | MEDRONE 2mg tablets | | | |
| fe61. | | | PREDNISOLONE 1mg tablets | | | |

**Supplementary Table S4: Secondary care code definitions and medications using the International Statistical Classification of Diseases and Related Health Problems (10^th^ revision)**

| **ICD-10 Code** | **Description** |
| --- | --- |
| **Kidney Disease** |  |
| [N00](https://www.icd10data.com/ICD10CM/Codes/N00-N99/N00-N08/N00-) | Acute nephritic syndrome |
| [N01](https://www.icd10data.com/ICD10CM/Codes/N00-N99/N00-N08/N01-) | Rapidly progressive nephritic syndrome |
| [N02](https://www.icd10data.com/ICD10CM/Codes/N00-N99/N00-N08/N02-) | Recurrent and persistent hematuria |
| [N03](https://www.icd10data.com/ICD10CM/Codes/N00-N99/N00-N08/N03-) | Chronic nephritic syndrome |
| [N04](https://www.icd10data.com/ICD10CM/Codes/N00-N99/N00-N08/N04-) | Nephrotic syndrome |
| [N05](https://www.icd10data.com/ICD10CM/Codes/N00-N99/N00-N08/N05-) | Unspecified nephritic syndrome |
| [N06](https://www.icd10data.com/ICD10CM/Codes/N00-N99/N00-N08/N06-) | Isolated proteinuria with specified morphological lesion |
| [N07](https://www.icd10data.com/ICD10CM/Codes/N00-N99/N00-N08/N07-) | Hereditary nephropathy, not elsewhere classified |
| [N08](https://www.icd10data.com/ICD10CM/Codes/N00-N99/N00-N08/N08-) | Glomerular disorders in diseases classified elsewhere |
| [N10](https://www.icd10data.com/ICD10CM/Codes/N00-N99/N10-N16/N10-) | Acute pyelonephritis |
| [N11](https://www.icd10data.com/ICD10CM/Codes/N00-N99/N10-N16/N11-) | Chronic tubulo-interstitial nephritis |
| [N12](https://www.icd10data.com/ICD10CM/Codes/N00-N99/N10-N16/N12-) | Tubulo-interstitial nephritis, not specified as acute or chronic |
| [N13](https://www.icd10data.com/ICD10CM/Codes/N00-N99/N10-N16/N13-) | Obstructive and reflux uropathy |
| [N14](https://www.icd10data.com/ICD10CM/Codes/N00-N99/N10-N16/N14-) | Drug- and heavy-metal-induced tubulo-interstitial and tubular conditions |
| [N15](https://www.icd10data.com/ICD10CM/Codes/N00-N99/N10-N16/N15-) | Other renal tubulo-interstitial diseases |
| [N16](https://www.icd10data.com/ICD10CM/Codes/N00-N99/N10-N16/N16-) | Renal tubulo-interstitial disorders in diseases classified elsewhere |
| [N17](https://www.icd10data.com/ICD10CM/Codes/N00-N99/N17-N19/N17-) | Acute kidney failure |
| [N18](https://www.icd10data.com/ICD10CM/Codes/N00-N99/N17-N19/N18-) | Chronic kidney disease (CKD) |
| [N19](https://www.icd10data.com/ICD10CM/Codes/N00-N99/N17-N19/N19-) | Unspecified kidney failure |
| [N20](https://www.icd10data.com/ICD10CM/Codes/N00-N99/N20-N23/N20-) | Calculus of kidney and ureter |
| [N21](https://www.icd10data.com/ICD10CM/Codes/N00-N99/N20-N23/N21-) | Calculus of lower urinary tract |
| [N22](https://www.icd10data.com/ICD10CM/Codes/N00-N99/N20-N23/N22-) | Calculus of urinary tract in diseases classified elsewhere |
| [N23](https://www.icd10data.com/ICD10CM/Codes/N00-N99/N20-N23/N22-) | Unspecified renal colic |
| [N25](https://www.icd10data.com/ICD10CM/Codes/N00-N99/N25-N29/N25-) | Disorders resulting from impaired renal tubular function |
| [N26](https://www.icd10data.com/ICD10CM/Codes/N00-N99/N25-N29/N26-) | Unspecified contracted kidney |
| [N27](https://www.icd10data.com/ICD10CM/Codes/N00-N99/N25-N29/N27-) | Small kidney of unknown cause |
| [N28](https://www.icd10data.com/ICD10CM/Codes/N00-N99/N25-N29/N28-) | Other disorders of kidney and ureter, not elsewhere classified |
| [N29](https://www.icd10data.com/ICD10CM/Codes/N00-N99/N25-N29/N29-) | Other disorders of kidney and ureter in diseases classified elsewhere |
| **Infections** |  |
|  |  |

| **Upper respiratory tract infections** | |  |  |  |  |
| --- | --- | --- | --- | --- | --- |
| J02.0 | Streptoccocal sore throat |  |  |  |  |
| J00 | Acute nasopharyngitis (common cold) |  |  |  |  |
| J01.90 | Acute sinusitis, unspecified |  |  |  |  |
| J02.9 | Acute phartngitis |  |  |  |  |
| J03.90 | Acute tonsilitis |  |  |  |  |
| J06.9 | Acute upper respiratory infections of unspecified site |  |  |  |  |
| J31.0 | Chronic rhinitis |  |  |  |  |
| J32.9 | Unspecified sinusitis (chronic) |  |  |  |  |
| R05 | Cough |  |  |  |  |
| **Lower respiratory tract infections** | |  |  |  |  |
| A37.90 | Whooping cough, unpecified organism |  |  |  |  |
| B97.4 | Respiratory syncytial virus |  |  |  |  |
| J41.0 | Simple chronic bronchitis |  |  |  |  |
| J41.1 | Micropurulent chronic bronchitis |  |  |  |  |
| J41.8 | Mixed simple and micropurulent chronic bronchitis |  |  |  |  |
| J42.0 | Unspecified chronic bronchitis |  |  |  |  |
| J14 | Pneumonia due to Haemophilus influenzae |  |  |  |  |
| J15.0 | Pneumonia due to Klebsiella pneumoniae |  |  |  |  |
| J15.1 | Pneumonia due to Pseudomonas |  |  |  |  |
| J15.2 | Pneumonia due to staphylococcus |  |  |  |  |
| J15.3 | Pneumonia due to streptococcus, group B |  |  |  |  |
| J15.4 | Pneumonia due to other streptococci |  |  |  |  |
| J15.5 | Pneumonia due to Escherichia coli |  |  |  |  |
| J15.6 | Pneumonia due to other aerobic Gram-negative bacteria |  |  |  |  |
| J15.7 | Pneumonia due to Mycoplasma pneumoniae |  |  |  |  |
| J15.8 | Other bacterial pneumonia |  |  |  |  |
| J15.9 | Bacterial pneumonia, unspecified |  |  |  |  |
| J18.0 | Bronchopneumonia, unspecified |  |  |  |  |
| J18.1 | Lobar pneumonia, unspecified |  |  |  |  |
| J18.2 | Hypostatic pneumonia, unspecified |  |  |  |  |
| J18.8 | Other pneumonia, organism unspecified |  |  |  |  |
| J18.9 | Pneumonia, unspecified |  |  |  |  |
| R09.1 | Pleurisy |  |  |  |  |
| **Tuberculosis** | |  |  |  |  |
| A15.0 | Tuberculosis of lung, confirmed by sputum microscopy with or without culture |  |  |  |  |
| A15.1 | Tuberculosis of lung, confirmed by culture only |  |  |  |  |
| A15.2 | Tuberculosis of lung, confirmed histologically |  |  |  |  |
| A15.3 | Tuberculosis of lung, confirmed by unspecified means |  |  |  |  |
| A15.4 | Tuberculosis of intrathoracic lymph nodes, confirmed bacteriologically and histologically |  |  |  |  |
| A15.5 | Tuberculosis of larynx, trachea and bronchus, confirmed bacteriologically and histologically |  |  |  |  |
| A15.6 | Tuberculous pleurisy, confirmed bacteriologically and histologically |  |  |  |  |
| A15.7 | Primary respiratory tuberculosis, confirmed bacteriologically and histologically |  |  |  |  |
| A15.8 | Other respiratory tuberculosis, confirmed bacteriologically and histologically |  |  |  |  |
| A15.9 | Respiratory tuberculosis unspecified, confirmed bacteriologically and histologically |  |  |  |  |
| A16.2 | Tuberculosis of lung, without mention of bacteriological or histological confirmation |  |  |  |  |
| A16.3 | Tuberculosis of intrathoracic lymph nodes, without mention of bacteriological or histological confirmation |  |  |  |  |
| A16.4 | Tuberculosis of larynx, trachea and bronchus, without mention of bacteriological or histological confirmation |  |  |  |  |
| A16.5 | Tuberculous pleurisy, without mention of bacteriological or histological confirmation |  |  |  |  |
| A16.7 | Primary respiratory tuberculosis without mention of bacteriological or histological confirmation |  |  |  |  |
| A16.8 | Other respiratory tuberculosis, without mention of bacteriological or histological confirmation |  |  |  |  |
| A16.9 | Respiratory tuberculosis unspecified, without mention of bacteriological or histological confirmation |  |  |  |  |
| A17.0 | Tuberculous meningitis |  |  |  |  |
| A17.1 | Meningeal tuberculoma |  |  |  |  |
| A17.8 | Other tuberculosis of nervous system |  |  |  |  |
| A17.9 | Tuberculosis of nervous system, unspecified |  |  |  |  |
| A18.0 | Tuberculosis of bones and joints |  |  |  |  |
| A18.1 | Tuberculosis of genitourinary system |  |  |  |  |
| A18.2 | Tuberculous peripheral lymphadenopathy |  |  |  |  |
| A18.3 | Tuberculosis of intestines, peritoneum and mesenteric glands |  |  |  |  |
| A18.4 | Tuberculosis of skin and subcutaneous tissue |  |  |  |  |
| A18.5 | Tuberculosis of eye |  |  |  |  |
| A18.6 | Tuberculosis of ear |  |  |  |  |
| A18.7 | Tuberculosis of adrenal glands |  |  |  |  |
| A18.8 | Tuberculosis of other specified organs |  |  |  |  |
| A19.0 | Acute miliary tuberculosis of a single specified site |  |  |  |  |
| A19.1 | Acute miliary tuberculosis of multiple sites |  |  |  |  |
| A19.2 | Acute miliary tuberculosis, unspecified |  |  |  |  |
| A19.8 | Other miliary tuberculosis |  |  |  |  |
| A19.9 | Miliary tuberculosis, unspecified |  |  |  |  |
| **GI infections** | |  |  |  |  |
| A03.0 | Shigellosis due to Shigella dysenteriae |  |  |  |  |
| A03.1 | Shigellosis due to Shigella flexneri |  |  |  |  |
| A03.2 | Shigellosis due to Shigella boydii |  |  |  |  |
| A03.3 | Shigellosis due to Shigella sonnei |  |  |  |  |
| A03.8 | Other shigellosis |  |  |  |  |
| A03.9 | Shingellosis, unspecified |  |  |  |  |
| A07.1 | Giardiasis |  |  |  |  |
| A00.0 | Cholera due to Vibrio cholerae 01, biovar cholerae |  |  |  |  |
| A00.1 | Cholera due to Vibrio cholerae 01, biovar eltor |  |  |  |  |
| A00.9 | Cholera, unspecified |  |  |  |  |
| A01.0 | Typhoid fever |  |  |  |  |
| A01.1 | Paratyphoid fever A |  |  |  |  |
| A01.2 | Paratyphoid fever B |  |  |  |  |
| A01.3 | Paratyphoid fever C |  |  |  |  |
| A01.4 | Paratyphoid fever, unspecified |  |  |  |  |
| A09.0 | Other and unspecified gastroenteritis and colitis of infectious origin |  |  |  |  |
| A09.9 | Gastroenteritis and colitis of unspecified origin |  |  |  |  |
| A04.0 | Enteropathogenic Escherichia coli infection |  |  |  |  |
| A04.1 | Enterotoxigenic Escherichia coli infection |  |  |  |  |
| A04.2 | Enteroinvasive Escherichia coli infection |  |  |  |  |
| A04.3 | Enterohaemorrhagic Escherichia coli infection |  |  |  |  |
| A04.4 | Other intestinal Escherichia coli infections |  |  |  |  |
| A04.5 | Campylobacter enteritis |  |  |  |  |
| A04.6 | Enteritis due to Yersinia enterocolitica |  |  |  |  |
| A04.7 | Enterocolitis due to Clostridium difficile |  |  |  |  |
| A04.8 | Other specified bacterial intestinal infections |  |  |  |  |
| A04.9 | Bacterial intestinal infection, unspecified |  |  |  |  |
| B98.0 | Helicobacter pylori [H.pylori] as the cause of diseases classified to other chapters |  |  |  |  |
| **Meningitis** | |  |  |  |  |
| A39.0 | Meningococcal meningitis |  |  |  |  |
| A39.1 | Waterhouse-Friderichsen syndrome |  |  |  |  |
| A39.2 | Acute meningococcaemia |  |  |  |  |
| A39.3 | Chronic meningococcaemia |  |  |  |  |
| A39.4 | Meningococcaemia, unspecified |  |  |  |  |
| A39.5 | Meningococcal heart disease |  |  |  |  |
| A39.8 | Other meningococcal infections |  |  |  |  |
| A39.9 | Meningococcal infection, unspecified |  |  |  |  |
| **Sepsis** |  |  |  |  |  |
| A40.0 | Sepsis due to streptococcus, group A |  |  |  |  |
| A40.1 | Sepsis due to streptococcus, group B |  |  |  |  |
| A40.2 | Sepsis due to streptococcus, group D |  |  |  |  |
| A40.3 | Sepsis due to Streptococcus pneumoniae |  |  |  |  |
| A40.8 | Other streptococcal sepsis |  |  |  |  |
| A40.9 | Streptococcal sepsis, unspecified |  |  |  |  |
| A41.0 | Sepsis due to Staphylococcus aureus |  |  |  |  |
| A41.1 | Sepsis due to other specified staphylococcus |  |  |  |  |
| A41.2 | Sepsis due to unspecified staphylococcus |  |  |  |  |
| A41.3 | Sepsis due to Haemophilus influenzae |  |  |  |  |
| A41.4 | Sepsis due to anaerobes |  |  |  |  |
| A41.5 | Sepsis due to other Gram-negative organisms |  |  |  |  |
| A41.8 | Other specified sepsis |  |  |  |  |
| A41.9 | Sepsis, unspecified |  |  |  |  |
| T81.44% | Sepsis following procedure |  |  |  |  |
| Severe sepsis with sepsis shock | |  |  |  |  |
| **Genitourinary infections** |  |  |  |  |  |
| N10 | Acute tubulo-interstitial nephritis |  |  |  |  |
| N11.0 | Nonobstructive reflux-associated chronic pyelonephritis |  |  |  |  |
| N11.1 | Chronic obstructive pyelonephritis |  |  |  |  |
| N11.8 | Other chronic tubulo-interstitial nephritis |  |  |  |  |
| N11.9 | Chronic tubulo-interstitial nephritis, unspecified |  |  |  |  |
| N12 | Tubulo-interstitial nephritis, not specified as acute or chronic |  |  |  |  |
| N16.0 | Renal tubulo-interstitial disorders in infectious and parasitic diseases classified elsewhere |  |  |  |  |
| N30.0 | Acute cystitis |  |  |  |  |
| N30.1 | Interstitial cystitis (chronic) |  |  |  |  |
| N30.2 | Other chronic cystitis |  |  |  |  |
| N30.3 | Trigonitis |  |  |  |  |
| N30.4 | Irradiation cystitis |  |  |  |  |
| N30.8 | Other cystitis |  |  |  |  |
| N30.9 | Cystitis, unspecified |  |  |  |  |
| N39.0 | Urinary tract infection, site not specified |  |  |  |  |
| **Joint infection** | | | | | |
| M00% | Pyogenic arthritis |  |  |  |  |
| M01% | Direct infections of joint in infectious and parasitic diseases classified elsewhere |  |  |  |  |
| M02% | Postinfective and reactive arthropathies |  |  |  |  |
| **Skin infection** | |  |  |  |  |
| B95.6% | Staphylococcus aureus as the cause of diseases classfied elsewhere |  |  |  |  |
| L03.0% | Cellulitis and acute lymphangitis of finger and toe |  |  |  |  |
| L03.1% | Cellulitis and acute lymphangitis of other parts of limb |  |  |  |  |
| L03.2% | Cellulitis and acute lymphangitis of face and neck |  |  |  |  |
| L03.3% | Cellulitis and acute lymphangitis of trunk |  |  |  |  |
| L03.8% | Cellulitis and acute lymphanitis of other sites |  |  |  |  |
| L03.9% | Cellulitis and acute lymphanitis, unspecified |  |  |  |  |
| L08.8% | Other specified local infection of the skin and subcutaneous tissue |  |  |  |  |
| L08.9 | Local infection of the skin and subcutaneous tissue, unpec |  |  |  |  |

**Supplementary Table S5: Orthopaedic surgery as indicated by OPCS-4 intervention and procedure codes from secondary care datasets**

| **OPCS-4 Code** | **OPCS-4 Term** |
| --- | --- |
| **W37** | **Monk total replacement of hip joint using cement** |
| W370 | Convrs from cement tot hip rep Conversion from cemented total hip replacement |
| W371 | Primary cemented tot hip repl Primary cemented total hip replacement |
| W372 | Conv to cemented tot hip repl Conversion to cemented total hip replacement |
| W373 | Revsion cemented total hip rep Revision cemented total hip replacement |
| W378 | Tot prosth repl hip + cem OS Total prosthetic replacement of hip joint using cement OS |
| W379 | Tot prosth repl hip + cem NOS Total prosthetic replacement of hip joint using cement NOS |
| **W38** | **Lord total hip replace no cem Lord total replacement of hip joint not using cement** |
| W380 | Conv from uncement tot hip rep Conversion from uncemented total hip replacement |
| W381 | Pry uncmnt tot hip replacement Primary uncemented total hip replacement |
| W382 | Cnvrs to uncmnt tot hip rplcmn Conversion to uncemented total hip replacement |
| W383 | Revisn uncemented tot hip rep Revision uncemented total hip replacement |
| W388 | Tot prosth repl hip no cem OS Total prosthetic replacement hip joint not using cement OS |
| W389 | Tot prosth repl hip no cem NOS Total prosthetic replacement hip joint not using cement NOS |
| **W39** | **Other total prosth repl hip Other total prosthetic replacement of hip joint** |
| W390 | Removal prev tot hip repl NEC Removal previous total prosthetic replacement hip joint NEC |
| W391 | Pry hybrid tot hip rplcmnt NEC Primary hybrid total hip replacement NEC |
| W392 | Cnvrs to hbrd tot hip rplc NEC Conversion to hybrid total hip replacement NEC |
| W393 | Revisn hybrid tot hip rep NEC Revision hybrid total hip replacement NEC |
| W394 | Attntn to tot hip rplcmnt NEC Attention to total hip replacement NEC |
| W398 | Total prosthet replace hip OS Other specified total prosthetic replacement of hip joint |
| W399 | Total prosthet replace hip NOS Total prosthetic replacement of hip joint NOS |
| **W40** | **Shiers total knee replacement Shiers total replacement of knee joint using cement** |
| **W40-W87** | **Knee joint operations** |
| W400 | Con from cemented tot knee rep Conversion from cemented total knee replacement |
| W401 | Pry cemented total knee replac Primary cemented total knee replacement |
| W402 | Conv to cemented tot knee repl Conversion to cemented total knee replacement |
| W403 | Revision cemented tot knee rep Revision cemented total knee replacement |
| W408 | Tot prosth repl knee + cem OS Total prosthetic replacement of knee joint using cement OS |
| W409 | Tot prosth repl knee + cem NOS Total prosthetic replacement of knee joint using cement NOS |
| W41 | Arthroplasty knee no cement Arthroplasty of knee joint not using cement |
| W410 | Removal prv uncem tot knee rep Removal previous uncemented total prosthet replacement knee |
| W411 | Primary uncmnt tot knee repl Primary uncemented total knee replacement |
| W412 | Cnvrs to uncmnt tot knee repl Conversion to uncemented total knee replacement |
| W413 | Revision uncmnt tot knee repl Revision uncemented total knee replacement |
| W418 | Tot pros repl knee no cem OS Total prosthetic replacement knee joint not using cement OS |
| W419 | Tot pros repl knee no cem NOS Total prosthetic replacement knee joint not using cement NOS |
| **W42** | **Other arthroplasty knee joint Other arthroplasty of knee joint** |
| W420 | Cnv fr hybrd tot knee repl NEC Conversion from hybrid total knee replacement NEC |
| W421 | Pry hybrid tot knee repl NEC Primary hybrid total knee replacement NEC |
| W422 | Con to hybrid tot knee rep NEC Conversion to hybrid total knee replacement NEC |
| W423 | Revi hybrid tot knee repl NEC Revision of hybrid total knee replacement NEC |
| W424 | Attention to tot knee repl NEC Attention to total knee replacement NEC |
| W428 | Other tot prosth knee repl OS Other total prosthetic replacement of knee joint OS |
| W429 | Other tot prosth knee rep NOS Other total prosthetic replacement of knee joint NOS |
| **W43** | **Prosth cmntd tot shldr rplcmnt Prosthetic cemented total shoulder replacement** |
| **W43-W45** | **Elbow joint operations** |
| **W43-W92** | **Other joint operations** |
| W430 | Cnvr fr cmnt tot shldr rplcmnt Conversion from cemented total shoulder replacement |
| W431 | Pry cmntd tot shldr replacmnt Primary cemented total shoulder replacement |
| W432 | Cnvr to cmntd tot shldr rplcmn Conversion to cemented total shoulder replacement |
| W433 | Rvsn cmntd tot shldr rplcmnt Revision cemented total shoulder replacement |
| W438 | Tot pros repl oth joint+cem OS Total prosthetic replacement of other joint using cement OS |
| W439 | Tot pr repl oth joint+cem NOS Total prosthetic replacement of other joint using cement NOS |
| **W44** | **Prsth uncmnt tot shldr rplcmnt Prosthetic uncemented total shoulder replacement** |
| W440 | Cnvr fr uncmnt tot shldr rplcm Conversion from uncemented total shoulder replacement |
| W441 | Pry uncmnt tot shldr replcmnt Primary uncemented total shoulder replacement |
| W442 | Cnvr to uncmnt tot shldr rplcm Conversion to uncemented total shoulder replacement |
| W443 | Rvsn uncmntd tot shldr rplcmnt Revision uncemented total shoulder replacement |
| W448 | Other joint repl no cement OS Other total prosthet replacem oth joint not using cement OS |
| W449 | Other joint repl no cement NOS Other total prosthet replacem oth joint not using cement NOS |
| **W45** | **Prosth hybrid tot shldr rplcmn Prosthetic hybrid total shoulder replacement** |
| W450 | Cnvr fr hybrid tot shldr rplcm Conversion from hybrid total shoulder replacement |
| W451 | Pry hybrid tot shldr rplcment Primary hybrid total shoulder replacement |
| W452 | Cnvr to hybrd tot shldr rplcmn Conversion to hybrid total shoulder replacement |
| W453 | Rvsn hybrid total shldr rplcmn Revision hybrid total shoulder replacement |
| W454 | Atten tot prosth joint rep NEC Attention to total prosthetic replacement of joint NEC |
| W454/Y037 | Rem prosth joint (no replace) Removal prosthesis from joint (no replacement) |
| W458 | Other tot prosth repl joint OS Other specified other total prosthetic replacement of joint |
| W459 | Other tot prosth rep joint NOS Other total prosthetic replacement of joint NOS |
| **W46** | **Austin-Moore hemiarthropl hip Austin - Moore hemiarthroplasty of hip joint using cement** |
| W460 | Removal prev cem repl hd femur Removal previous cemented prosthetic replacement head femur |
| W461 | Pry cmntd hemiarthroplasty hip Primary cemented hemiarthroplasty of hip |
| W462 | Cnvrs to cmnt hemiarthrpls hip Conversion to cemented hemiarthroplasty of hip |
| W463 | Rvsn cmntd hemiarthroplsty hip Revision cemented hemiarthroplasty of hip |
| W468 | Oth sp prsth cmntd hemiart hip Other specified prosthetic cemented hemiarthroplasty of hip |
| W469 | Prsth cmnt hemiarthrpl hip NOS Prosthetic cemented hemiarthroplasty of hip NOS |
| **W47** | **Prsth uncmnt hemiarthrpl hip Prosthetic uncemented hemiarthroplasty of hip** |
| W470 | Cnvr fr uncmnt hemiarthrpl hip Conversion from uncemented hemiarthroplasty of hip |
| W471 | Pry uncmntd hemiarthropl hip Primary uncemented hemiarthroplasty of hip |
| W472 | Cnvrs to uncmnt hemiarthrp hip Conversion to uncemented hemiarthroplasty of hip |
| W473 | Rvsn uncmnt hemiarthrpl hip Revision uncemented hemiarthroplasty of hip |
| W478 | Other pros uncem hemiarthr hip Other specified prosthetic uncemented hemiarthroplasty hip |
| W479 | Prsth uncmn hemiarthrp hip NOS Prosthetic uncemented hemiarthroplasty of hip NOS |
| **W48** | **Other arthroplasty head femur Other arthroplasty of head of femur** |
| W480 | Cnvr fr prev hemiarthr hip NEC Conversion from previous hemiarthroplasty of hip NEC |
| W481 | Pry prosth hemiarthrpl hip NEC Primary prosthetic hemiarthroplasty of hip NEC |
| W482 | Cnvr to prsth hemiarth hip NEC Conversion to prosthetic hemiarthroplasty of hip NEC |
| W483 | Rvsn prsth hemiarthrpl hip NEC Revision of prosthetic hemiarthroplasty of hip NEC |
| W484 | Atn to prsth hemiarthr hip NEC Attention to prosthetic hemiarthroplasty of hip NEC |
| W488 | Oth sp oth prsth hemiarthr hip Other specified other prosthetic hemiarthroplasty of hip |
| W489 | Oth prosth hemiarthrpl hip NOS Other prosthetic hemiarthroplasty of hip NOS |
| **W49** | **Pros replacem head hum + cemnt Prosthetic replacement of head of humerus using cement** |
| **W49-W51** | **Shoulder joint operations** |
| W490 | Cnvrsn fr cmnt hemiarthr shldr Conversion from cemented hemiarthroplasty of shoulder |
| W491 | Primary cmntd hemiarthr shldr Primary cemented hemiarthroplasty of shoulder |
| W492 | Cnvrs to cmntd hemiarthr shldr Conversion to cemented hemiarthroplasty of shoulder |
| W493 | Rvsn cmntd hemiarthr shldr Revision cemented hemiarthroplasty of shoulder |
| W498 | Oth spec cmntd hemiarthr shldr Other specified cemented hemiarthroplasty of shoulder |
| W499 | Prosth cmnt hemiarth shldr NOS Prosthetic cemented hemiarthroplasty of shoulder NOS |
| **W50** | **Prosth uncmntd hemiarthr shldr Prosthetic uncemented hemiarthroplasty of shoulder** |
| W500 | Cnvr fr uncmnt hemiarth shldr Conversion from uncemented hemiarthroplasty of shoulder |
| W501 | Primary uncmnt hemiarthr shldr Primary uncemented hemiarthroplasty of shoulder |
| W502 | Cnvr to uncmnt hemiarthr shldr Conversion to uncemented hemiarthroplasty of shoulder |
| W503 | Rvsn uncmntd hemiarthr shldr Revision uncemented hemiarthroplasty of shoulder |
| W508 | Oth spec uncmnt hemiarth shldr Other specified uncemented hemiarthroplasty of shoulder |
| W509 | Pros uncmntd hemiart shldr NOS Prosthetic uncemented hemiarthroplasty of shoulderNOS |
| **W51** | **Other prosth repl head humerus Other prosthetic replacement of head of humerus** |
| W510 | Conv from hyb hemrth shoul NEC Conversion from hemiarthroplasty of shoulder NEC |
| W511 | Prim hybr hemiarth should NEC Primary hybrid hemiarthroplasty of shoulder NEC |
| W512 | Conv-hybr hemiarth should NEC Conversion to hybrid hemiarthroplasty of shoulder NEC |
| W513 | Revis hyb hemiarth should NEC Revision hybrid hemiarthroplasty of shoulder NEC |
| W514 | Atten replace head humerus NEC Attention to prosthetic replacement of head of humerus NEC |
| W518 | Other prosth repl head hum OS Other prosthetic replacement of head of humerus OS |
| W519 | Oth prsth replcmnt hd hum NOS Other prosthetic replacement of head of humerus NOS |
| **W52** | **Cmntd unicmprtmntl kn rplcmnt Cemented unicompartmental knee replacement** |
| W520 | Cnvr fr cmnt unicmprt kn rplcm Conversion from cemented unicompartmental knee replacement |
| W521 | Pry cmntd unicmprtmn kn rplcmn Primary cemented unicompartmental knee replacement |
| W522 | Cnvr to cmnt unicmpr kn rplcmn Conversion to cemented unicompartmental knee replacement |
| W523 | Rvsn cmnt unicmprtmn kn rplcmn Revision cemented unicompartmental knee replacement |
| W528 | Prosth repl oth artic + cem OS Prosthetic replacement articulation oth bone using cement OS |
| W529 | Pros repl oth artic + cem NOS Prosthetic replacement articulatn oth bone using cement NOS |
| **W53** | **Uncmnt unicmprtmnt kn rplcmnt Uncemented unicompartmental knee replacement** |
| W530 | Cnvr fr uncmn unicmprt kn rplc Conversion from uncemented unicompartmental knee replacement |
| W531 | Prmy uncmnt unicmprt kn rplcmn Primary uncemented unicompartmental knee replacement |
| W532 | Cnvr to uncmn unicmprt kn rplc Conversion to uncemented unicompartmental knee replacement |
| W533 | Rvsn uncmnt unicmprtm kn rplcm Revision uncemented unicompartmental knee replacement |
| W538 | Prosth repl articul no cem OS Prosthet replacement articulat oth bone not using cement OS |
| W539 | Prosth repl articul no cem NOS Prosthet replacement articulat oth bone not using cement NOS |
| **W54** | **Hybrid unicomprtmnt kn rplcmnt Hybrid unicompartmental knee replacement** |
| W540 | Cnvr fr hybrd unicmprt kn rplc Conversion from hybrid unicompartmental knee replacement |
| W541 | Pry hybrid unicmprtm kn rplcmn Primary hybrid unicompartmental knee replacement |
| W542 | Cnvr to hybrd unicmpr kn rplcm Conversion to hybrid unicompartmental knee replacement |
| W543 | Rvsn hybrd unicmprtmt kn rplcm Revision hybrid unicompartmental knee replacement |
| W544 | Atten repl articul bone NEC Attention to prosthetic replacement of articulation NEC |
| W548 | Other repl artic oth bone OS Other prosthetic replacement of articulation of oth bone OS |
| W549 | Other repl artic oth bone NOS Other prosthetic replacement of articulation of oth bone NOS |
| **W55** | **Prosth interposit arthroplasty Prosthetic interposition arthroplasty** |
| W550 | Con proth intrpstn arthrpls Conversion from previous prosth interposition arthroplasty |
| W551 | Pry proth interpstn arthrplsty Primary prosthetic interposition arthroplasty |
| W552 | Rvsn proth interpstn arthrplsy Revision of prosthetic interposition arthroplasty |
| W553 | Cnvr to prth intrpstn arthrpls Conversion to prosthetic interposition arthroplasty |
| W554 | Atn prth intrpstn arthrpls NEC Attention to prosthetic interposition arthroplasty NEC |
| W558 | Oth sp prth intrpstn arthrplsy Other specified prosthetic interposition arthroplasty |
| W559 | Proth intrpstn arthroplast NOS Prosthetic interposition arthroplasty NOS |
| **W56** | **Other interposit arthroplasty Other interposition arthroplasty** |
| W560 | Con prev intrpstn arthrpls NEC Conversion from previous interposition arthroplasty NEC |
| W561 | Prim interpos arth MTP joint Primary interposit arthroplasty metatarsophalang joint NEC |
| W562 | Pry interpstn arthroplasty NEC Primary interposition arthroplasty NEC |
| W563 | Rvsn of intrpstn arthrplst NEC Revision of interposition arthroplasty NEC |
| W564 | Cnvr to intrpstn arthrplst NEC Conversion to interposition arthroplasty NEC |
| W568 | Oth sp interpstn recnstrctn jt Other specified interposition reconstruction of joint |
| W569 | Interposit reconstr joint NOS Interposition reconstruction of joint NOS |
| **W57** | **Excision arthroplasty** |
| W570 | Cnvr fr prev excsn arthroplsty Conversion from previous excision arthroplasty |
| W571 | Prim exc arthropl 1st MTP join Primary excision arthroplasty 1st metatarsophalangeal joint |
| W572 | Pry excision arthroplasty NEC Primary excision arthroplasty NEC |
| W572/Z843 | Excision arthroplasty of hip |
| W573 | Rvsn of excision arthroplasty Revision of excision arthroplasty |
| W574 | Cnvrsn to excisn arthroplasty Conversion to excision arthroplasty |
| W578 | Oth sp excsn reconstrctn of jt Other specified excision reconstruction of joint |
| W579 | Exc reconstruction joint NOS Excision reconstruction of joint NOS |
| **W58** | **Other arthroplasty** |
| W580 | Conv fr prev resurf arthroplas Conversion from previous resurfacing arthroplasty of joint |
| W581 | Prim resurfacing arthroplasty Primary resurfacing arthroplasty of joint |
| W582 | Rvsn of resurfcng arthroplasty Revision of resurfacing arthroplasty |
| W588 | Reconstruct joint c free flap Reconstruction of joint with free flap |
| W589 | Other reconstruction joint NOS Other reconstruction of joint NOS |
| **W59** | **Fusion of other toe joint** |
| **W59 -W79** | **Foot joint operations** |
| W591 | Pry arthrodesis 1st MTPJ & rep Primary arthrodesis 1st MTPJ & replace lesser MTPJ |
| W592 | Pry arthrodesis 1st MTPJ & exc Primary arthrodesis 1st MTPJ & excision lesser MTPJ |
| W593 | Fusion of first MTP joint Fusion of first metatarsophalangeal joint of toe |
| W594 | Pry arthrdsis IPJ great toe Primary arthrodesis interphalangeal joint of great toe |
| W595 | Pry arthrdsis IPJ oth toe NEC Primary arthrodesis of interphalangeal joint other toe NEC |
| W596 | Revision arthrdsis toe joint Revision arthrodesis of toe joint |
| W598 | Fusion of first MTP joint OS Other specified fusion of first metatarsophalangeal joint |
| W599 | Fusion of other toe joint NOS |
| **W60** | **Fuse joint extraart bone graft Fusion of other joint and extraarticular bone graft** |
| W600 | Cnvrs fr extrartic arthrod NEC Conversion from extraarticular arthrodesis NEC |
| W601 | Pry extraart arthrodsis NEC Primary extraarticular arthrodesis of joint NEC |
| W602 | Rvsn extrartic arthrodesis NEC Revision extraarticular arthrodesis NEC |
| W603 | Conv to extrart arthrodsis NEC Conversion to extraarticular arthrodesis NEC |
| W608 | Fuse joint & extraart graft OS Fusion of joint and extraarticular bone graft OS |
| W609 | Fuse joint & extraart graf NOS Fusion of joint and extraarticular bone graft NOS |
| **W61** | **Other fuse joint & artic graft Fusion of other joint and other articular bone graft** |
| W610 | Cnv fr prev intraart arthrdsis Conversion from previous intraarticular arthrodesis |
| W611 | Prim arthrod & artic graft NEC Primary arthrodesis and articular bone graft NEC |
| W612 | Rev arthrod & artic graft NEC Revision of arthrodesis and articular bone graft NEC |
| W613 | Conv to arthrod artic graf NEC Conversion to arthrodesis and articular bone graft NEC |
| W618 | Fuse joint & artic graft OS Other specified fusion of joint and articular bone graft |
| W619 | Fuse joint & artic graft NOS Fusion of joint and articular bone graft NOS |
| **W62** | **Other primary fusion of joint** |
| W621 | Prim arthrodes & int fixat NEC Primary arthrodesis and internal fixation of joint NEC |
| W622 | Prim arthrodes & ext fixat NEC Primary arthrodesis and external fixation of joint NEC |
| W628 | Other primary arthrodesis OS Other specified other primary fusion of joint |
| W629 | Simple arthrodesis |
| **W63** | **Revisional fusion of joint** |
| W631 | Revis arthrodes int fixat NEC Revision of arthrodesis and internal fixation NEC |
| W632 | Revis arthrodes ext fixat NEC Revision of arthrodesis and external fixation NEC |
| W638 | Revisional fusion of joint OS Other specified revisional fusion of joint |
| W639 | Revision of arthrodesis NEC |
| **W64** | **Conversion to arthrodesis NEC** |
| W640 | Conversion from prev arthrodes Conversion from previous arthrodesis NEC |
| W641 | Conv to arthrod & int fix NEC Conversion to arthrodesis and internal fixation NEC |
| W642 | Conv to arthrod & ext fix NEC Conversion to arthrodesis and external fixation NEC |
| **V22** | **Prim decompress cervical spine Primary decompression operation on cervical spine** |
| V22-V54 | Cerv and thorac spine ops OS Other specified operations on cervical or thoracic spine |
| V221 | Pry ant dcmpr cx spn crd+fsn Primary ant decompression of cervical spinal cord+fusion |
| V222 | Pry ant dcmprs cx spn crd NEC Primary anterior decompression of cervical spinal cord NEC |
| V223 | Prim foraminotomy cerv spine Primary foraminotomy of cervical spine |
| V228 | Prmry post decomprsn cerv cord Primary posterior decompression cervical cord |
| V229 | Prim decomp cervical spine NOS Primary decompression operation on cervical spine NOS |
| **V23** | **Revis decomp cervical spine Revisional decompression operations on cervical spine** |
| V231 | Rev ant dcmpr crv sp crd+fsn Revisional ant decompression op cervicl spinal cord+fusion |
| V232 | Rev ant decomp cerv cord NEC Revisional anterior decompression cervical spinal cord NEC |
| V233 | Revis foraminotomy cerv spine Revisional foraminotomy of cervical spine |
| V238 | Rev post decomprsn cerv cord Revision posterior decompression cervical cord |
| V239 | Rev decomp cervical spine NOS Revisional decompression of cervical spine NOS |
| **V24** | **Decompress thoracic spine NEC Decompression of thoracic spine NEC** |
| V241 | Prim decompr fusion thor spine Primary decompress thoracic spinal cord fusion thorac spine |
| V242 | Prim decompress thor spine NEC Primary decompression of thoracic spine NEC |
| V243 | Rvsnal post decomprs thor dsc Revisional posterior decompression of thoracic disc |
| V248 | Decompression thorac spine OS Other specified decompression of thoracic spine |
| V249 | Prmry post decompr thorac disc Primary posterior decompression of thoracic disc |
| **V25** | **Primary decomp lumbar spine op Primary decompression operations on lumbar spine** |
| V25-V39 | Lumbar spinal cord operations |
| V25-V54 | Lumbar spine operations OS Other specified operations on lumbar spine |
| V251 | Pry extn pst dcmprs lmb sp+fsn Primary extndd post decompression lumbar spine and fusion |
| V252 | Pry extnd pst dcmpr lmb sp NEC Primary extended posterior decompression lumbar spine NEC |
| V253 | Prmy post dcmprsn lmbr spn+fsn Primary posterior decompression lumbar spine and fusion |
| V254 | Prmy post dcmprsn lmbr spine Primary posterior decompression lumbar spine |
| V255 | Prim post decompr LS cord NEC Primary posterior decompression of lumbar spinal cord NEC |
| V256 | Prmy foraminotomy lmbr spine Primary foraminotomy of lumbar spine |
| V258 | Prim decompr op lumb spine OS Primary decompression operation on lumbar spine OS |
| V259 | Prim decompr op lumb spine NOS Primary decompression operation on lumbar spine NOS |
| **V26** | **Revis decompr ops lumbar spine Revisional decompression operations on lumbar spine** |
| V261 | Rev extnd pst dcmpr lmb sp+fsn Revision extndd post decompression lumbar spine and fusion |
| V262 | Rev extnd pst dcmpr lmb sp NEC Revision extended posterior decompression lumbar spine NEC |
| V263 | Rvsn post dcmprsn lmbr spn+fsn Revision posterior decompression lumbar spine and fusion |
| V264 | Rvsn post dcmprsn lmbr spine Revision posterior decompression lumbar spine |
| V265 | Revis post decomp LS cord NEC Revisional posterior decompression of lumbar spinal cord NEC |
| V266 | Rvsnal foraminotomy lmbr spine Revisional foraminotomy of lumbar spine |
| V268 | Revis decompr op lumbar sp OS Revisional decompression operation on lumbar spine OS |
| V269 | Revis decompr op lumb sp NOS Revisional decompression operation on lumbar spine NOS |
| **V27** | **Decompress unspecified spine Decompression operations on unspecified spine** |
| V27-V54 | Oth spine operations,site unsp Other spine operations, site unspecified |
| V271 | Prim decomp sp cord & fuse NEC Primary decompression of spinal cord & fusion spine jnt NEC |
| V272 | Prim decompr spinal cord NEC Primary decompression of spinal cord NEC |
| V273 | Revis decompr spinal cord NEC Revisional decompression of spinal cord NEC |
| V278 | Decompression of spine OS Other specified decompression of spine |
| V279 | Decompression of spine NOS |
| **V29** | **Primary excis cervical IV disc Primary excision of cervical intervertebral disc** |
| **V29-V54** | **Cerv+thoracic spine ops Cervical and thoracic spine operations** |
| V291 | Pry laminec excis cerv IV disc Primary laminectomy excision of cervical intervert disc |
| V292 | 1st hemilaminec cerv IV disc Primary hemilaminectomy excision of cervical IV disc |
| V293 | 1st fenestration cerv IV disc Primary fenestration excision of cervical intervert disc |
| V294 | Badgeley ant fusion cerv spine Badgeley anterior fusion of cervical spine |
| V295 | 1st ant excis cervic disc NEC Primary anterior excision of cervical intervertebr disc NEC |
| V296 | 1st cervical microdiscectomy Primary microdiscectomy of cervical intervertebral disc |
| V298 | Primary excis cervical disc OS Primary excision of cervical intervertebral disc OS |
| V299 | 1st post exc cervical IV disc Primary posterior excision of cervical intervertebral disc |
| **V30** | **Revis cervical disc excis ops Revisional excision of cervical intervertebral disc ops** |
| V301 | Revis laminec exc cerv IV disc Revisional laminectomy excision of cervical intervert disc |
| V302 | Rev hemilaminect exc cerv disc Revisional hemilaminectomy excision cervical intervert disc |
| V303 | Rev fenestration exc cerv disc Revisional fenestration excision of cervical intervert disc |
| V304 | Rvsn ant excsn cerv disc+fusn Revision anterior excision cervical disc and fusion |
| V305 | Revis ant excis cerv disc NEC Revisional anterior excision cervical intervert disc NEC |
| V306 | Revis cervical microdiscectomy Revisional microdiscectomy of cervical intervertebral disc |
| V308 | Revis post excis cerv disc NEC Revisional posterior excision of cervical intervert disc NEC |
| V309 | Revis cervic IV disc excis NOS Revisional excision of cervical intervertebral disc NOS |
| **V31** | **Excision of thoracic disc NEC Excision of thoracic intervertebral disc NEC** |
| V311 | Prmry ant excsn thor disc+fsn Primary anterior excision thoracic disc and fusion |
| V312 | Prim ant/lat exc thor disc NEC Primary anterolateral excision thoracic intervert disc NEC |
| V313 | Prim costotransversect th disc Primary costotransversectomy of thoracic intervertebral disc |
| V318 | Prim excis thoracic disc OS Primary excision of thoracic intervertebral disc OS |
| V319 | Prim excis thoracic disc NOS Primary excision of thoracic intervertebral disc NOS |
| **V32** | **Rvsn excs thr intrvrt dsc NEC Revisional excision of thoracic intervertebral disc NEC** |
| V321 | Rvsn ant excsn thor disc+fsn Revision anterior excision thoracic disc and fusion |
| V322 | Rev AL excis thoracic disc NEC Revisional anterolateral excision thorac intervert disc NEC |
| V323 | Rev costotransversec thor disc Revisional costotransversectomy thoracic intervertebral disc |
| V328 | Rvsn decomprs+fsn thorac spn Revision decompression and fusion thoracic spine |
| V329 | Revis excis thoracic disc NOS Revisional excision thoracic intervertebral disc NOS |
| **V33** | **Primary lumbar discectomy** |
| V331 | Prim laminect exc lumbar disc Primary laminectomy excision of lumbar intervertebral disc |
| V332 | Prim fenestration lumbar disc Primary fenestration of lumbar intervertebral disc |
| V333 | Prmy ant exc lmbr disc+fusion Primary anterior excision of lumbar disc and fusion |
| V334 | Prmy ant exc lmbr disc NEC Primary anterior excision of lumbar disc NEC |
| V335 | Prmy ant exc lmbr dsc+pst fsn Primary anterior excision of lumbar disc and posterior fusn |
| V336 | Pry ant exc lmb dsc+pst instrm Primary ant excision lumbar disc+post instrumentation |
| V337 | Primary lumbar microdiscectomy |
| V338 | Prmy post excision lumbar disc Primary posterior excision of lumbar disc |
| V339 | Primary lumbar discectomy NOS Primary excision of lumbar intervertebral disc NOS |
| **V34** | **Revisional lumbar discectomy** |
| V341 | Revis laminec exc lumbar disc Revisional laminectomy excision of lumbar intervert disc |
| V342 | Revis fenestr exc lumbar disc Revisional fenestration excision of lumbar intervert disc |
| V343 | Rvsn ant exc lmbr disc+fusion Revisional anterior excision of lumbar disc and fusion |
| V344 | Rvsn ant excisn lmbr disc NEC Revisional anterior excision of lumbar disc NEC |
| V345 | Rvs ant excs lmbr dsc+pst fusn Revisional anterior excision of lumbar disc and post fusion |
| V346 | Rev ant excs lmbrdsc+pst instr Revisional anterior excision lumbar disc + post instrument |
| V347 | Rvsnl lmbr microdiscectomy Revisional lumbar microdiscectomy |
| V348 | Rvsnl post excsn lmbr disc Revisional posterior excision of lumbar disc |
| V349 | Revision lumbar discectomy NOS Revisional excision of lumbar intervertebral disc NOS |
| **V35** | **Excis intervertebral disc NEC Excision of intervertebral disc NEC** |
| V351 | Primary excision IV disc NEC Primary excision of intervertebral disc NEC |
| V352 | Revision excision IV disc NEC Revisional excision of intervertebral disc NEC |
| V358 | Percutaneous discectomy |
| V359 | Excision intervertebr disc NOS Excision of intervertebral disc NOS |
| V359/Y081 | Laser discectomy |
| **V37** | **Prim fusion cervical spine jnt Primary fusion of joint of cervical spine** |
| V371 | Brooks fuse atlantoaxial joint Brooks fusion of atlantoaxial joint |
| V372 | Post fusion joint cx spine NEC Posterior fusion of joint of cervical spine NEC |
| V373 | Transoral fuse atlantoax joint Transoral fusion of atlantoaxial joint |
| V374 | Fusion of atlantooccipital jnt Fusion of atlantooccipital joint |
| V378 | Fusion atlantoaxial joint NEC Fusion of atlantoaxial joint NEC |
| V379 | Cervic spine joint fusion NOS Fusion of joint of cervical spine NOS |
| **V38** | **Oth prim fusion joint lumb sp Other primary fusion of joint of lumbar spine** |
| V381 | Prmry post fusion thorac spine Primary posterior fusion of thoracic spine |
| V382 | Prmry post fusn lmbr spine Primary posterior fusion of lumbar spine |
| V383 | Wiltse posterior fusion spine Wiltse posterior fusion of spine |
| V384 | Pry intrtrns fusn lmbr spn NEC Primary intertransverse fusion lumbar spine NEC |
| V388 | Primrposterolat fusn lmbr spn Primary posterolateral fusion lumbar spine |
| V389 | Prim fusion lumbar spine NOS Primary fusion of joint of lumbar spine NOS |
| **V39** | **Other revision lumbar fusion Other revisional fusion of joint of lumbar spine** |
| V391 | Revisnal fusion cerv spine Revisional fusion of cervical spine |
| V392 | Revis post fusion thorac spine Revisional posterior fusion of joint of thoracic spine |
| V393 | Rvsn post fusn intrlam lmbr sp Revision posterior interlaminar fusion of lumbar spine |
| V394 | Revis post lumbar fusion NEC Revisional posterior fusion of joint of lumbar spine NEC |
| V395 | Rvs intrtrnsv fsn lmbr spn NEC Revision intertransverse fusion of lumbar spine NEC |
| V398 | Rvsn posterolat fusn lmbr spne Revision posterolateral fusion lumbar spine |
| V399 | Revision of lumbar fusion NOS |
| **V41** | **Crct spn defrm+instrmntn Correction of spinal deformity and instrumentation** |
| V411 | Knodt spinal distraction rod Posterior attachment Knodt spinal distraction rod to spine |
| V412 | Crct spn defrm+ant instrmntn Correction of spinal deformity and anterior instrumentation |
| V413 | Removal Crctal spn instrmntn Removal correctional spinal instrumentation |
| V418 | Crct spn dfrm+instr+ped fxn sy Crctn spn deform+instrumnt with pedicular fixation system |
| V419 | Instrumental cor def spine NOS Instrumental correction deformity of spine NOS |
| **V42** | **Other corr deformity spine Other correction of deformity of spine** |
| V421 | Excision of rib hump |
| V422 | Epiphysiodesis of spine Epiphysiodesis of spine - deformity correction |
| V423 | Antrolat release spn defrm+gft Anterolateral release of spinal deformity and graft |
| V428 | Other corr deformity spine OS Other specified correction of deformity of spine |
| V429 | Other corr deformity spine NOS Correction of deformity of spine NOS |
| **V43** | **Extirpation spine lesion NEC Extirpation of lesion of spine NEC** |
| V431 | Excis lesion cervical vertebra Excision of lesion of cervical vertebra |
| V432 | Excis lesion thoracic vertebra Excision of lesion of thoracic vertebra |
| V433 | Excis lesion lumbar vertebra Excision of lesion of lumbar vertebra |
| V438 | Gill excis spondylolisthesis Gill excision of spondylolisthesis |
| V439 | Excision lesion of spine NEC Excision of lesion of spine NEC |
| **V44** | **Spine fracture decompression Decompression of fracture of spine** |
| V441 | Complex decompress # spine Complex decompression of fracture of spine |
| V442 | Anterior decompression # spine Anterior decompression of fracture of spine |
| V443 | Posterior decompress # spine Posterior decompression of fracture of spine |
| V448 | Spine fracture decompress OS Other specified decompression of fracture of spine |
| V449 | Spine fracture decompress NOS Decompression of fracture of spine NOS |
| **V45** | **Other spine fracture reduction Other reduction of fracture of spine** |
| V451 | Open reduct exc facet # spine Open reduction of fracture of spine & excis facet of spine |
| V452 | Open reduction # spine NEC Open reduction of fracture of spine NEC |
| V453 | Manipulative reduction # spine Manipulative reduction of fracture of spine |
| V458 | Spinal extension traction # Spinal extension traction for fracture of spine |
| V459 | Other spine fracture reduc NOS Other reduction of fracture of spine NOS |
| **V46** | **Fixation of fracture of spine** |
| V461 | Pry opn red spn #+int fix+plte Primary open reduc spinal fracture+internal fix+plate |
| V462 | Fixat # spine Harrington rod Fixation of fracture of spine using Harrington rod |
| V463 | Pry opn red spn #+int fix+wire Primary open reduc spinal fracture+internal fix+wire |
| V464 | Halo skull traction # spine Halo skull traction for fracture of spine |
| V468 | Pry op rd sp #+int fix+seg wre Primary open reduc spinal #+intern fix+segmental wire system |
| V469 | Fixation spine fracture NOS Fixation of fracture of spine NOS |
| **V52** | **Other intervertebral disc ops Other operations on intervertebral disc** |
| V521 | Enzyme destruct intervert disc Enzyme destruction of intervertebral disc |
| V522 | Destruction of disc NEC Destruction of intervertebral disc NEC |
| V523 | Discography intervert disc Discography of intervertebral disc |
| V524 | Prim ant/lat biops thorac disc Primary anterolateral biopsy of thoracic intervertebral disc |
| V528 | Other intervert disc op OS Other specified operation on intervertebral disc |
| V529 | Other intervert disc op NOS Operation on intervertebral disc NOS |
| **V54** | **Other ops on spine & vertebra Other ops on spine and vertebra** |
| V541 | Transoral excisn odontoid peg Transoral excision of odontoid peg |
| V542 | Graft of bone to spine NEC |
| V543 | Osteotomy of spine NEC |
| V544 | Injection into paraspinal area |
| V548 | Primary thoracic spine op Primary operation on thoracic spine |
| V549 | Primary thoracic spine op NOS Primary operation on thoracic spine NOS |
| **W01** | **Complex reconstruction thumb Complex reconstruction of thumb** |
| **W01-W05** | **Complex reconstruct hand+foot Complex reconstruction operations on hand and foot** |
| **W01-W92** | **Other bone & joint operations Other bone and joint operations** |
| W011 | Microvasc transf toe to thumb Microvascular transfer of toe to thumb |
| W012 | Pollicisation of finger |
| W013 | Thmb rcn usng bne grft+skn flp Thumb reconstruction using bone graft and skin flap |
| W014 | Thmb recon usng bne lngth proc Thumb reconstruction using bone lengthening procedure |
| W015 | Opponensplasty thumb |
| W018 | Free phalangeal transfer thumb Free phalangeal transfer to thumb |
| W019 | Complex reconstruct thumb NOS Complex reconstruction of thumb NOS |
| **W02** | **Other complex reconstr of hand Other complex reconstruction of hand** |
| W021 | Proximal row carpectomy |
| W022 | Metacarpal supp op on carpus Metacarpal support operation on carpus |
| W023 | Multiple jnt reconstr hand NEC Multiple joint reconstruction of hand NEC |
| W024 | Cmplx soft tiss recons hnd NEC Complex soft tissue reconstruction in hand NEC |
| W028 | Reconstruction hand local flap Reconstruction of hand with local flap |
| W029 | Other complex reconst hand NOS Other complex reconstruction of hand NOS |
| **W03** | **Complex reconstr of forefoot Complex reconstruction of forefoot** |
| W031 | Kessel reconstruction forefoot Kessel reconstruction of forefoot |
| W032 | Helal metatarsal osteotomy |
| W033 | Total correction of claw toe |
| W034 | Robert Jones proced great toe Robert Jones procedure great toe |
| W035 | Fusion joints mid & forefoot Localised fusion of joints of midfoot and forefoot |
| W038 | Correction of metatarsus varus |
| W039 | Complex reconstr forefoot NOS Complex reconstruction of forefoot NOS |
| **W04** | **Complex reconstr of hindfoot Complex reconstruction of hindfoot** |
| W041 | Local fusion hindfoot joints Localised fusion of joints of hindfoot |
| W042 | Dunn triple fusion of foot |
| W043 | Goldthwait hindfoot stabilise Goldthwait stabilisation of hindfoot |
| W044 | Muscle strip from os calcis Stripping of muscle from os calcis |
| W045 | Exc lat wedge & fusion os calc Rel medial soft tiss hindfoot & exc lat wedge fusion os calc |
| W048 | Articular fusion subtalar jnt Articular fusion subtalar joint |
| W049 | Complex reconstr hindfoot NOS Complex reconstruction of hindfoot NOS |
| **W05** | **Prosthetic replacement of bone** |
| W051 | Articul prosthet replace bone Articulated prosthetic replacement of bone |
| W058 | Prosthet replacement bone OS Other specified prosthetic replacement of bone |
| W059 | Prosthet replacement bone NOS Prosthetic replacement of bone NOS |
| **W06** | **Excision of entire bone** |
| W06-W36 | Bone operations |
| W061 | Total excision of cervical rib |
| W062 | Total excision of rib NEC |
| W063 | Total excision of patella |
| W064 | Total excision of sesamoid NEC Total excision of sesamoid bone NEC |
| W065 | Talectomy |
| W066 | Total excision of coccyx |
| W068 | Total excision of bone OS Other specified total excision of bone |
| W069 | Ostectomy NEC |
| W069+W059 | Tot excis & prosthet rep bone Total excision of bone and prosthetic replacement for bone |
| **W07** | **Excision of ectopic bone** |
| W071 | Excision of cross union bone Excision of cross union of bone |
| W072 | Excis periartic ectopic bone Excision of periarticular ectopic bone |
| W073 | Excis intramusc ectopic bone Excision of intramuscular ectopic bone |
| W078 | Excision of ectopic bone OS Other specified excision of ectopic bone |
| W079 | Excision of ectopic bone NOS |
| **W08** | **Other excision of bone** |
| W081 | Excision of tuberosity of bone |
| W082 | Excision of overgrowth of bone |
| W083 | Excision of bony excrescence Excision of excrescence of bone |
| W084 | Excision of fragment of bone |
| W085 | Excision metatarsal head NEC Excision of head of metatarsal bone NEC |
| W085+Z814 | Rttr cuff dcomp-opn acrmplsty Rotator cuff decompression - open acromioplasty |
| W088 | Excision of synostosis |
| W089 | Other excision of bone NOS Excision of bone NOS |
| W089+W051 | Excis bone & art pros rep NEC Excision of bone and articulated prosthetic replacement NEC |
| W089+W059 | Excis bone & prosth repl NEC Excision of bone and prosthetic replacement NEC |
| W089+W329 | Excis bone & bone graft HFQ Excision of bone and bone graft however further qualified |
| **W09** | **Extirpation of lesion of bone** |
| W091 | Excision of lesion of bone |
| W092 | Curett bone lesion & graft HFQ Curettage of lesion of bone and graft HFQ |
| W093 | Curettage lesion of bone NEC Curettage of lesion of bone NEC |
| W094 | Destructn lesion of bone NEC Destruction of lesion of bone NEC |
| W098 | Cryoablation of bone lesion |
| W099 | Extirpation bone lesion NOS Extirpation of lesion of bone NOS |
| **W10** | **Open osteoclasis** |
| W101 | Op osteocl ang cor int fix HFQ Open osteoclasis, angular correction & internal fixation HFQ |
| W102 | Op osteocl ang cor ext fix HFQ Open osteoclasis angular correction & external fixation HFQ |
| W103 | Angulatory osteotomy NEC |
| W104 | Open osteoclasis & int fix NEC Open osteoclasis and internal fixation NEC |
| W105 | Open osteoclasis+ext fxtn NEC Open osteoclasis and external fixation NEC |
| W108 | Rotational osteotomy |
| W109 | Open surgical fracture NOS Open surgical fracture of bone NOS |
| **W11** | **Other surgical bone fracture Other surgical fracture of bone** |
| W111 | Closed osteoclasis |
| W118 | Other surg fracture of bone OS Other specified other surgical fracture of bone |
| W119 | Other surg fracture bone NOS Other surgical fracture of bone NOS |
| **W12** | **Angulation periartic osteotomy Angulation periarticular osteotomy** |
| W121 | Bios ang peri ost int fix HFQ Biosseus angulation periarticul osteotomy & int fixation HFQ |
| W122 | Ang peri osteot & int fix NEC Angulation periarticular osteotomy and internal fixation NEC |
| W123 | Bios ang peri ost ext fix HFQ Biosseus angulation periarticul osteotomy & ext fixation HFQ |
| W124 | Ang peri osteot & ext fix NEC Angulation periarticular osteotomy and external fixation NEC |
| W125 | Biosseus ang peri osteot NEC Biosseus angulation periarticular osteotomy NEC |
| W128 | Akin's osteotomy |
| W129 | Angulat periartic osteot NOS Angulation periarticular division of bone NOS |
| **W13** | **Other periarticular osteotomy** |
| W131 | Rotation periarticular osteot Rotation periarticular osteotomy |
| W132 | Displacement osteotomy |
| W133 | Cuneiform osteotomy |
| W138 | Other periarticular osteot OS Other specified other periarticular division of bone |
| W139 | Other periarticular osteot NOS Other periarticular division of bone NOS |
| **W14** | **Diaphyseal division of bone** |
| W141 | Ang diaph osteot & int fix HFQ Angulation diaphyseal osteotomy and internal fixation HFQ |
| W142 | Ang diaph osteot & ext fix HFQ Angulation diaphyseal osteotomy and external fixation HFQ |
| W143 | Angulation diaphys osteot NEC Angulation diaphyseal osteotomy NEC |
| W144 | Rotat diaph ost & int fix HFQ Rotation diaphyseal osteotomy and internal fixation HFQ |
| W145 | Rotat diaph ost & ext fix HFQ Rotation diaphyseal osteotomy and external fixation HFQ |
| W146 | Rotation diaphys osteotomy NEC Rotation diaphyseal osteotomy NEC |
| W148 | Diaphyseal division of bone OS Other specified diaphyseal division of bone |
| W149 | Diaphyseal division bone NOS Diaphyseal division of bone NOS |
| **W15** | **Osteotomy of bone of foot** |
| W151 | Mitchell hallux valgus osteot Mitchell osteotomy for hallux valgus |
| W152 | Golden hallux valgus operation Golden osteotomy base 1st metatarsal bone for hallux valgus |
| W153 | Hallux valgus osteotomy NEC |
| W154 | Osteotomy head metatarsal Osteotomy of head of metatarsal |
| W155 | Osteotomy of midfoot tarsal |
| W158 | Oth spec osteotomy foot bone Other specified osteotomy of bone of foot |
| W159 | Osteotomy of bone of foot NOS |
| **W16** | **Other division of bone** |
| W161 | Multip osteot & int fix HFQ Multiple osteotomy and internal fixation HFQ |
| W162 | Multip osteot & ext fix HFQ Multiple osteotomy and external fixation HFQ |
| W163 | Multiple osteotomy NEC |
| W164 | Intn fixation of osteotomy NEC Internal fixation of osteotomy NEC |
| W165 | Extn fixation of osteotomy NEC External fixation of osteotomy NEC |
| W168 | Other division of bone OS Other specified other division of bone |
| W169 | Osteotomy NEC |

**Supplementary Table S6: Hazard ratio of factors associated with being treated with non-IL6 bDMARD first (Univariate analysis).**

|  | Hazard ratio | 95% Confidence Interval | P-value |
| --- | --- | --- | --- |
| Female | 1.21 | 1.09 to 1.36 | 0.001* |
| Increasing age at diagnosis | 0.98 | 0.98 to 0.99 | <0.001* |
| Disease duration | 1.17 | 1.14 to 1.19 | <0.001* |
| Increasing BMI | 0.98 | 0.96 to 0.99 | 0.04* |
| Living in a rural area | 1.04 | 0.91 to 1.18 | 0.606 |
| Ever smoked | 0.96 | 0.82 to 1.21 | 0.613 |
| Each additional DMARD | 1.51 | 1.43 to 1.59 | <0.001* |
| Infections (pre-treatment) | 0.85 | 0.75 to 0.96 | 0.009* |
| Orthopaedic surgery (pre-treatment) | 1.45 | 1.31 to 1.61 | <0.001* |
| Kidney disease (pre-treatment) | 0.31 | 0.25 to 0.38 | <0.001* |
| Hyperlipidaemia (pre-treatment) | 0.66 | 0.55 to 0.78 | <0.001* |
| Hypertension (pre-treatment) | 0.67 | 0.60 to 0.74 | <0.001* |
| Diabetes (pre-treatment) | 0.62 | 0.53 to 0.74 | <0.001* |
| Cardiovascular disease (pre-treatment) | 0.73 | 0.63 to 0.84 | <0.001* |
| Steroid use | 1.27 | 1.13 to 1.42 | <0.001* |

**p<0.05*

**Supplementary Table S7: Hazard ratio of factors associated with being treated with IL-6 bDMARD first (Univariate analysis).**

|  | Hazard ratio | 95% Confidence Interval | P-value |
| --- | --- | --- | --- |
| Female | 1.16 | 0.76 to 1.77 | 0.490 |
| Increasing age at diagnosis | 0.98 | 0.97 to 0.99 | 0.002* |
| Disease duration | 0.94 | 0.88 to 1.00 | 0.051 |
| Increasing BMI | 1.06 | 0.99 to 1.13 | 0.059 |
| Living in a rural area | 0.99 | 0.59 to 1.67 | 0.985 |
| Ever smoked | 1.12 | 0.65 to 1.92 | 0.689 |
| Each additional DMARD | 1.43 | 1.16 to 1.77 | 0.001* |
| Infections (pre-treatment) | 1.73 | 1.15 to 2.59 | 0.008* |
| Orthopaedic surgery (pre-treatment) | 1.08 | 0.72 to 1.61 | 0.715 |
| Kidney disease (pre-treatment) | 1.65 | 1.04 to 2.60 | 0.032* |
| Hyperlipidaemia (pre-treatment) | 1.01 | 0.57 to 1.81 | 0.961 |
| Hypertension (pre-treatment) | 0.69 | 0.46 to 1.02 | 0.064 |
| Diabetes (pre-treatment) | 1.31 | 0.78 to 2.19 | 0.305 |
| Cardiovascular disease (pre-treatment) | 0.59 | 0.32 to 1.09 | 0.094 |
| Steroid use | 1.11 | 0.73 to 1.69 | 0.631 |

**p<0.05*

**Supplementary Table S8: Hazard ratio of factors associated with treatment failure in non-IL bDMARD treated patients (univariate)**

|  | Hazard ratio | 95% Confidence Interval | P-value |
| --- | --- | --- | --- |
| Female | 1.14 | 0.92 to 1.43 | 0.230 |
| Increasing age at diagnosis | 0.99 | 0.98 to 0.99 | <0.001* |
| Disease duration | 1.00 | 0.96 to 1.04 | 0.887 |
| Increasing BMI | 1.02 | 0.97 to 1.06 | 0.460 |
| Living in a rural area | 0.90 | 0.70 to 1.16 | 0.417 |
| Ever smoked | 0.94 | 0.71 to 1.26 | 0.703 |
| Each additional DMARD | 1.07 | 0.97 to 1.19 | 0.195 |
| Infections (pre-treatment) | 0.80 | 0.62 to 1.04 | 0.091 |
| Infections (post-treatment) | 1.23 | 0.89 to 1.70 | 0.216 |
| Orthopaedic surgery (pre-treatment) | 0.82 | 0.67 to 0.99 | 0.042* |
| Orthopaedic surgery (post-treatment) | 1.11 | 0.81 to 1.52 | 0.502 |
| Kidney disease (pre-treatment) | 1.00 | 0.63 to 1.59 | 0.987 |
| Hyperlipidaemia (pre-treatment) | 0.92 | 0.65 to 1.31 | 0.641 |
| Hypertension (pre-treatment) | 0.89 | 0.73 to 1.09 | 0.262 |
| Diabetes (pre-treatment) | 1.16 | 0.83 to 1.61 | 0.337 |
| Cardiovascular disease (pre-treatment) | 0.90 | 0.67 to 1.20 | 0.461 |
| Steroid use | 1.63 | 1.27 to 2.10 | <0.001 |

**p<0.05*

**Supplementary Table S9: Hazard ratio of factors associated with treatment failure in IL-6 bDMARD treated patients (univariate)**

|  | Hazard ratio | 95% Confidence Interval | P-value |
| --- | --- | --- | --- |
| Female | 1.26 | 0.42 to 3.78 | 0.677 |
| Increasing age at diagnosis | 1.00 | 0.96 to 1.30 | 0.782 |
| Disease duration | 1.07 | 0.82 to 1.38 | 0.682 |
| Increasing BMI | 0.99 | 0.85 to 1.16 | 0.907 |
| Living in a rural area | 0.61 | 0.14 to 2.62 | 0.504 |
| Ever smoked | 1.25 | 0.36 to 4.26 | 0.725 |
| Each additional DMARD | 1.00 | 0.59 to 1.67 | 0.991 |
| Infections (pre-treatment) | 1.57 | 0.62 to 3.93 | 0.340 |
| Infections (post-treatment) | 1.44 | 0.42 to 4.92 | 0.559 |
| Orthopaedic surgery (pre-treatment) | 1.07 | 0.43 to 2.69 | 0.883 |
| Orthopaedic surgery (post-treatment) | 0.64 | 0.77 to 5.33 | 0.679 |
| Kidney disease (pre-treatment) | 1.76 | 0.68 to 4.60 | 0.246 |
| Hyperlipidemia (pre-treatment) | 1.57 | 0.46 to 5.35 | 0.474 |
| Hypertension (pre-treatment) | 1.88 | 0.78 to 4.52 | 0.160 |
| Diabetes (pre-treatment) | 0.85 | 0.25 to 2.92 | 0.801 |
| Cardiovascular disease (pre-treatment) | 1.77 | 0.52 to 6.05 | 0.361 |
| Steroid use | 1.01 | 0.37 to 2.78 | 0.982 |

**Supplementary Table S10: Patient profiles of the bDMARD experienced rheumatoid arthritis patients taking second line of treatment of either non-IL-6 or anti-IL-6 bDMARDs**

|  | Non-anti-IL-6 bDMARDs initiated  (n= 298) | Anti-IL-6 bDMARDs initiated  (n= 87) | Difference (95% Confidence Interval) |
| --- | --- | --- | --- |
| Mean age diagnosis in years (SD) | 57.3 (12.0) | 55.4 (12.1) | 1.9 (-1.0 to 4.8) |
| Mean disease duration, years (SD) | 8.1 (2.3) | 8.4 (2.2) | 0.3 (-0.9 to 0.3) |
| Female, % (n) | 76.5% (228) | 80.5% (70) | 4.0 (-12.6 to 6.6) |
| Body Mass Index, % (n) | 27.3 (6.2) | 29.0 (6.6) | 1.7 (-4.5 to 1.1) |
| Ever smoked, % (n) | 13.1% (39) | 6.9% (6) | 6.2 (-1.9 to 11.9) |
| Biologic treatment change/fail, % (n) | 13.1% (39) | 41.4% (36) | 28.3 (17.6 to 39.3)* |
| Infections pre-treatment, % (n) | 14.4% (43) | 12.6% (11) | 1.8 (-7.5 to 8.8) |
| Infections post-treatment, % (n) | 92.3% (275) | 90.8% (79) | 1.5 (-4.3 to 9.8) |
| Orthopaedic surgery pre-treatment, % (n) | 33.9% (101) | 33.3% (29) | 0.6 (-11.1 to 11.1) |
| Orthopaedic surgery post-treatment, % (n) | 17.8% (53) | 25.3% (22) | 7.5 (-1.8 to 18.3) |
| Diabetes pre-treatment, % (n) | 9.1% (27) | 8.1% (7) | 10 (-7.1 to 6.6) |
| Hyperlipidaemia pre-treatment, % (n) | 7.1% (21) | 9.2% (8) | 2.1 (-10.4 to 3.5) |
| Hypertension pre-treatment, % (n) | 28.5% (85) | 32.2% (28) | 3.7 (-15.1 to 6.7) |
| Cardiovascular disease pre-treatment, % (n) | 13.1% (39) | <5 | - |
| Steroid use, % (n) | 81.9% (244) | 89.7% (78) | 7.8 (-12.9 to 14.6) |

**p<0.05 (SD: Standard Deviation; bDMARD: biologic disease-modifying anti-rheumatic drug; IL-6: interleukin-6 )*

**Supplementary Table S11: Hazard ratio of factors associated with treatment failure in non-biologic naïve biologic treated patients (Univariate analysis).**

|  | Hazard ratio | 95% Confidence Interval | P-value |
| --- | --- | --- | --- |
| Female | 1.13 | 0.64 to 2.00 | 0.666 |
| Increasing age at diagnosis | 1.00 | 0.98 to 1.02 | 0.891 |
| Disease duration | 1.05 | 0.93 to 1.18 | 0.434 |
| Increasing BMI | 1.00 | 0.90 to 1.10 | 0.925 |
| Living in a rural area | 0.61 | 0.29 to 1.27 | 0.184 |
| Ever smoked | 0.58 | 0.25 to 1.33 | 0.200 |
| Each additional DMARD | 0.79 | 0.59 to 1.04 | 0.095 |
| Hospitalised for infection (pre-treatment) | 1.13 | 0.60 to 2.15 | 0.704 |
| Hospitalised for infection (post-treatment) | 0.66 | 0.32 to 1.38 | 0.275 |
| Orthopaedic surgery (pre-treatment) | 1.02 | 4.11 to 2.54 | 0.962 |
| Orthopaedic surgery (post-treatment) | 2.94 | 0.72 to 12.0 | 0.133 |
| Kidney disease (pre-treatment) | 1.44 | 0.53 to 3.06 | 0.476 |
| Hyperlipidaemia (pre-treatment) | 1.06 | 0.46 to 2.45 | 0.884 |
| Hypertension (pre-treatment) | 1.40 | 0.87 to 2.26 | 0.163 |
| Diabetes (pre-treatment) | 0.89 | 0.39 to 2.05 | 0.780 |
| Cardiovascular disease (pre-treatment) | 0.53 | 0.21 to 1.30 | 0.166 |
| Steroid use | 1.22 | 0.62 to 2.37 | 0.567 |
